# Cis‐Regulation of an m^6^A Eraser by an Insertion Variant Associated with Survival of Patients With Non‐Small Cell Lung Carcinoma

**DOI:** 10.1002/advs.202407652

**Published:** 2024-12-16

**Authors:** Lei Cheng, Qiangsheng Hu, Yanan Wang, Wei Nie, Haijiao Lu, Bo Zhang, Genming Zhao, Shiyun Ding, Feng Pan, Yinchen Shen, Runbo Zhong, Ruoxin Zhang

**Affiliations:** ^1^ Department of Respiratory and Critical Care Medicine Shanghai Chest Hospital Shanghai Jiaotong University School of Medicine Huaihai West Road No.241 Shanghai 200030 China; ^2^ Department of Thoracic Surgery Shanghai Pulmonary Hospital Tongji University School of Medicine Shanghai 200433 China; ^3^ Department of Medical Oncology The Affiliated Hospital of Qingdao University Qingdao Shandong 266000 China; ^4^ Department of Epidemiology School of Public Health Key Laboratory of Public Health Safety Ministry of Education Fudan University Shanghai 200032 China; ^5^ Yiwu Research Institute of Fudan University Yiwu Zhejiang 322000 China; ^6^ Cancer Institute Fudan University Shanghai Cancer Center Department of Oncology Shanghai Medical College Fudan University Shanghai 200032 China

**Keywords:** Cis‐regulation, genetic variants, m^6^A pathway, NSCLC, survival

## Abstract

N6‐methyladenosine (m^6^A) serves as one of the crucial RNA modifications for genes involved in cancer progression. Here, 7273 expression quantitative trait loci potentially regulating 30 m6A pathway genes are identified from the GTEx database, with 69 single nucleotide polymorphisms significantly associated with survival of non‐small cell lung carcinoma (NSCLC) patients (*n =* 1523) from the ongoing genome‐wide association study after false positive probability tests. Notably, the rs151198415 locus, situated in a potential enhancer region, demonstrated a prolonged survival effect with the C>CCACG insertion, which is validated in an independent prospective cohort (*n =* 237), yielding a pooled hazard ratio of 0.72 (*p* = 0.007). Mechanistically, the rs151198415 C>CCACG insertion engaged in long‐range interaction with the promoter of m^6^A eraser *ALKBH5*, promoting *ALKBH5* transcription by the creation of an EGR1 binding site. Then, *ALKBH5* upregulated *FBXL5* expression by m^6^A demethylation, which is dependent on the ALKBH5 H204 amino acid site and specific m^6^A sites on *FBXL5* mRNA. Finally, the ALKBH5‐FBXL5 axis reduces intracellular reactive oxygen species levels, leading to PI3K/AKT and NF‐kB pathway inhibition and consequently suppresses NSCLC proliferation and metastasis in vitro and in vivo. Triggered by an insertion variant, this remote cis‐regulation of m^6^A eraser and the downstream molecular events modulate the survival of NSCLC patients.

## Graphical abstract

A common insertion variant exhibits cis‐regulation potential for the m6A eraser *ALKBH5*, which demethylates FBXL5 mRNA, leading to reduction in reactive oxygen species levels, inhibition of PI3K/AKT and NF‐kB pathways, and consequent suppression of NSCLC cells. This functional variant may serve as a predictive biomarker for the survival of non‐small cell lung cancer patients.

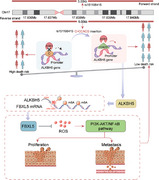

## 1 Background

In recent years, lung cancer continues to be one of the most diagnosed and leading causes of cancer‐related mortality worldwide, particularly in China. According to 2020 cancer statistics in China,^[^ ^1^ ^]^ lung cancer was the most common type of cancer, with a death rate of ≈28.16 per 100 000, accounting for ≈30% of cancer‐related deaths.^[^ ^1^ ^]^

Non‐small cell lung carcinoma (NSCLC) accounts for ≈85% of lung cancers.^[^ ^2^ ^]^ Optimal treatment of lung cancer at an early stage could significantly improve the survival of the patients, and investigating biomarkers for survival prediction in the early stage may benefit the treatment decision for NSCLC patients. In addition to traditional risk factors for prognosis, mounting evidence suggested that inherited factors such as germline single nucleotide polymorphisms (SNPs) are also involved in lung cancer progression. The non‐invasiveness of this type of biomarker makes it a promising tool for survival prediction in NSCLC patients.^[^ ^3^, ^4^, ^5^ ^]^ Our team has been focusing on genetic variants and cancer patients’ survival over the past few years and reported survival predictive variants in DNA repair genes,^[^ ^6^ ^]^ mTOR pathway,^[^ ^7^ ^]^ human leukocyte antigens loci,^[^ ^8^ ^]^ and other related genes.^[^ ^9^ ^]^ In addition, mechanistically, we also revealed the interplay between genetic variants and microRNAs,^[^ ^7^ ^]^ as well as long‐range interactions between genetic variants and promoters of the target genes,^[^ ^8^, ^9^ ^]^ all of which highlighted the vital roles of genetic variants in cancer epigenetic regulations and survival modulation.

N6‐methyladenosine (m^6^A) methylation has been demonstrated to be one of the most common posttranscriptional RNA modifications known to affect lung cancer progression.^[^ ^10^ ^]^ Previous studies reported that m^6^A‐mediated RNA modification in NSCLC is typically regulated by m^6^A writer, eraser, and reader proteins.^[^ ^11^ ^]^ However, these studies primarily focused on the downstream genes regulated by m^6^A modification,^[^ ^12^, ^13^, ^14^ ^]^ with few comprehensively assessing the role of genetic variants in m^6^A pathway modulation and their biological impact on the survival of NSCLC patients. To our knowledge, only one recent study has demonstrated a genetic variant with the potential to affect m^6^A modification in NSCLC survival. They investigated the underlying mechanism for the adverse impact of a germline mutation on the survival of the patients who received EGFR tyrosine kinase inhibitors.^[^ ^15^ ^]^ It provided new insights into the relationship between m^6^A modification and NSCLC patients’ survival, highlighting the need for a comprehensive assessment of the role of genetic variants in m^6^A pathway genes and their impact on the survival modulation of NSCLC patients. In the present study, we systematically reviewed 7273 genetic variants with the potential to modulate the expression of 30 genes in the m^6^A pathway. After quality control, we assessed the association of these genetic variants with the survival of NSCLC patients and revealed the molecular mechanism underlying the observed association, thus addressing a novel axis on the m^6^A network modulation by genetic variants in NSCLC.

## 2 Results

### 2.1 eQTL Analysis of m^6^A Pathway Genes and Survival Associations in NSCLC Patients

**Figure 1:**
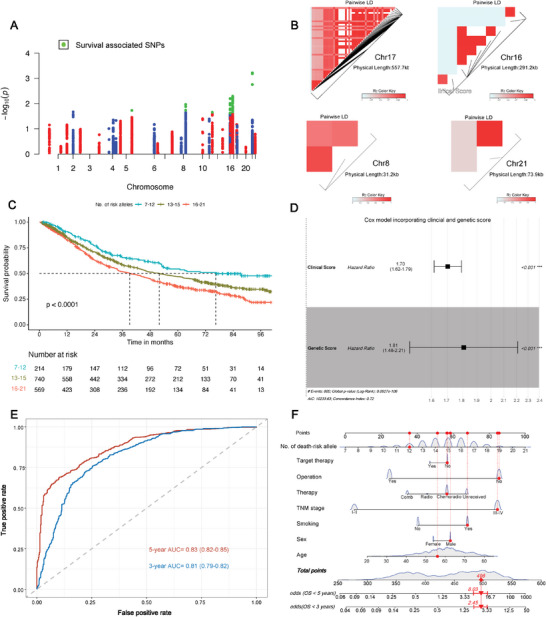
Survival association of genetic variants that may regulate m^6^A pathway genes in 1523 NSCLC patients. We included 7273 genetic variants with eQTL effect on m^6^A pathway genes from the GTEx database, among which 6325 were eligible for survival association in 1523 NSCLC patients from our FUSCC cohort after quality control. A) As shown by the Manhattan plot, 69 SNPs (green dot) were identified as significant survival‐associated SNPs and passed FPRP and BFDP false positive test; B) Linkage disequilibrium plots for 69 survival‐associated SNPs across chromosomes 17, 16, 8, and 21; A total of 11 tag SNPs were identified from the 69 survival‐associated SNPs. C) Death‐risk alleles of the 11 tag SNPs demonstrated significant association with NSCLC patients’ survival in a dose‐dependent manner; Cox models were constructed using clinical variables and the 11 tag SNPs, respectively, to generate weighed clinical and genetic scores. D) Survival associations of two types of scores were performed by the Cox regression model, with hazard ratio and 95% CI presented in the forest plot; E) Combining genetic and clinical scores yielded substantial predictive ability for survival of 1523 NSCLC patients, as demonstrated by survival ROC; F) Considering both clinical and genetic variables, we constructed a nomogram for survival prediction applicable in clinical practice. The red vertical line and numbers were an example for generating 3‐year/5‐year odds of death under specified clinical/genetic characterization using this nomogram.

Using the GTEx dataset, we identified 7273 eQTL variants with regulatory potential for 30 m^6^A pathway genes in multiple tissues (Table S8, Supporting Information). After quality control, 6325 genetic variants were included for further survival analysis in our FUSCC lung cancer GWAS study consisting of 1523 NSCLC patients (Table S9, Supporting Information). Finally, we identified 69 significant survival‐associated SNPs in NSCLC patients (*p* < 0.05, FPRP<0.2, BFDP<0.8), as visualized in the Manhattan plot in **Figure** **1**A. These survival‐associated variants are mostly located on chromosome 17 (*n =* 52), followed by chromosome 16 (*n =* 9), chromosome 8 (*n =* 3), chromosome 21 (*n =* 3), chromosome 5 (*n =* 1), and chromosome 14 (*n =* 1). We then plotted the LD plot which revealed considerable LD for survival‐associated SNPs (Figure 1B). To obtain a simplified survival prediction model, we further identified 11 tag SNPs for survival association analysis in combination. We found poorer survival and increased death risk with an increased number of the risk alleles (Figure 1C, Table S10, Supporting Information). We then selected clinical variables, including age, sex, smoking status, TNM stage, and treatment to construct clinical scores weighed by their coefficients in the Cox regression model. Weighted genetic score was established by including the 11 tag SNPs in the Cox model. We found the hazard ratio was comparable between the clinical (HR = 1.70, 95% CI = 1.62–1.79, *p* < 0.001, Figure 1D) and genetic score (HR = 1.81, 95% CI = 1.48–2.21, *p* < 0.001, Figure 1D). Combining the clinical and genetic scores resulted in substantial predictive ability for survival as indicated by the receiver operating characteristic (ROC) curve [3‐year area under curve (AUC) = 0.81, and 5‐year AUC = 0.83, Figure 1E). To facilitate potential clinical practice for survival prediction, we established a nomogram by genetic and clinical variables to assess 3‐year and 5‐year survival probabilities (Figure 1F).

### 2.2 eQTL Validation and Functional Prediction for Survival‐Associated SNPs

**Figure 2:**
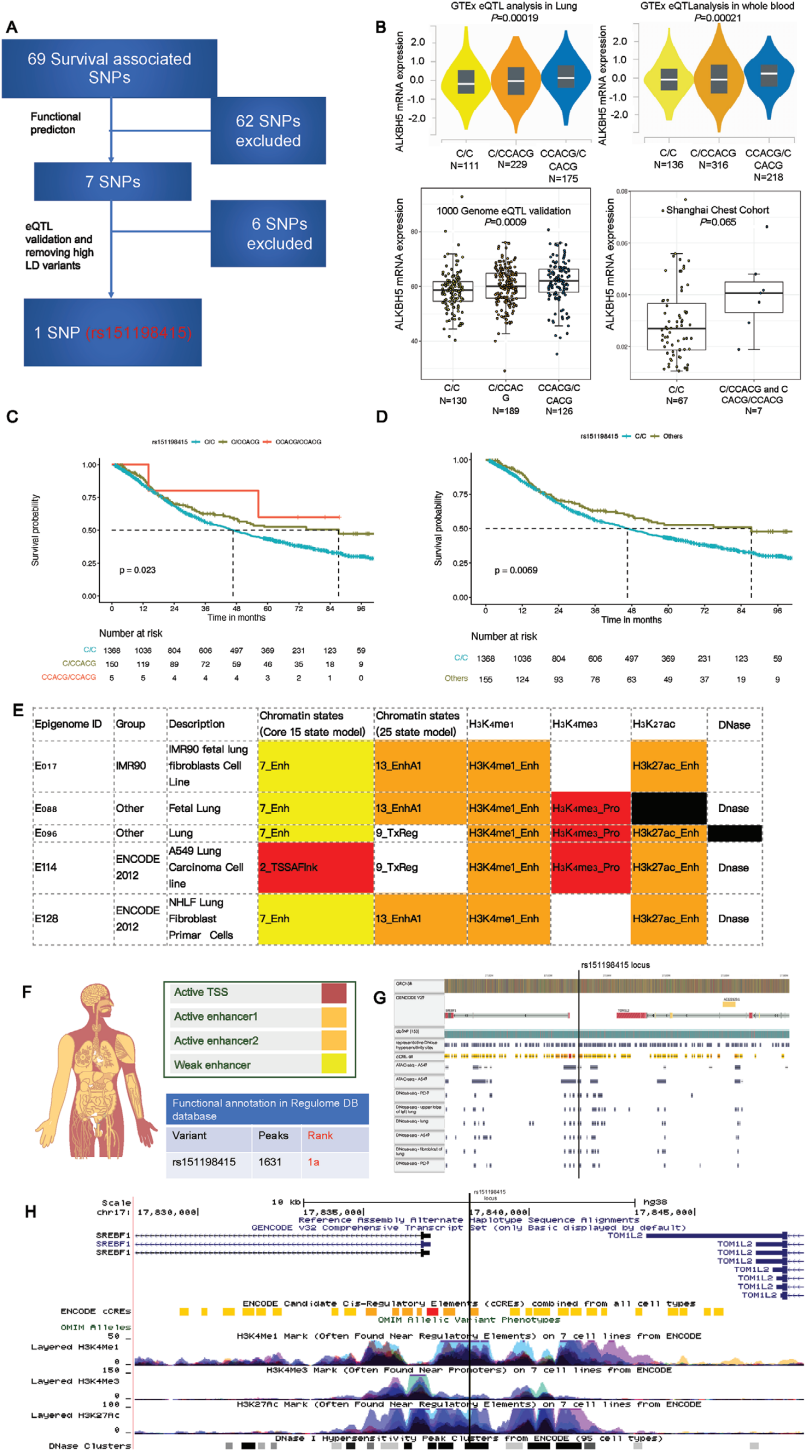
Functional prediction, genotype‐phenotype association, and survival association of the rs151198415 variant. A) After functional prediction, eQTL validation, and removal of high LD variants, the rs151198415 variant was ultimately identified as a potentially functional variant from the 69 survival‐associated SNPs; B) The rs151198415 C>CCACG change upregulated *ALKBH5* mRNA expression, as evidenced by 515 lung tissues and 670 whole blood samples in the GTEx database, a finding further validated in 445 lymphoblastoid cell lines from the 1000 Genomes project. We also collected 74 NSCLC tissue samples and found a borderline significant association of the rs151198415 C>CCACG change with *ALKBH5* upregulation; C) Kaplan–Meier curves demonstrate that the rs151198415 C>CCACG change prolongs survival of 1523 NSCLC patients, shown in both additive and D) dominant models; E) Analysis from the Haploreg database suggests enhancer involvement of the rs151198415 locus in lung tissues and A549 NSCLC cells, supported by ChIP enrichment of histone markers including H3K27ac and H3K4me1. DNase‐seq further indicated the chromatin accessibility in the rs151198415 region; F) Merged organ‐specific functional annotations from the Regulome DB database also suggested enhancer function for the rs151198415 variant specifically in lung, G) with additional DNase‐seq and ATAC‐seq evidence in lung tissues and A549 cells visualized by Regulome DB; H) Histone markers in 7 different cell lines (merged) visualized from the ENCODE database, which suggested the enhancer and cis‐regulatory function of the rs151198415 locus.

With the goal of investigating the functional SNP in the survival‐associated SNPs identified above, we conducted in silico functional prediction and eQTL validation as depicted in **Figure** **2**A, a process which discovered rs151198415 as the only eQTL and potential functional variant for further analysis. In brief, the functions of 69 survival‐associated SNPs were predicted by the Regulome DB database, leaving 7 potentially functional SNPs with Regulome DB scores 1a‐b (Figure 2A; Table S11, Supporting Information). Further eQTL validation by 445 lymphoblastoid cell lines from the 1000G database resulted in 3 variants, including rs151198415 (Figure 2B), rs7359509, and rs2955371 (Figure S1, Supporting Information), which may modulate the mRNA expression of *ALKBH5*, an m^6^A eraser. However, we found notable LD among the 3 variants. Because the rs151198415 variant was ranked with the highest functional score (1a) by the Regulome DB database (Table S11, Supporting Information), it was selected from the 3 variants for further functional analysis. As indicated by GTEx multi‐tissue eQTL plot for rs151198415‐*ALKBH5* regulation (Figure S2A, Supporting Information) and our summary (Figure S2B, Supporting Information), the results were significant in 23 tissues (46.94%) but were insignificant in 26 tissues (53.06%). Intriguingly, an upregulated expression of *ALKBH5* was observed in all significant results, and similar trends were seen in most of the insignificant results (84.62%). We visualized the significant GTEx eQTL effect in 515 lung tissues and 670 whole blood samples, and the validation by 445 lymphoblastoid cell lines in the 1000G project (Figure 2B). We also collected 74 NSCLC tissues in our hospital for this SNP‐gene correlation analysis and found a trend toward *ALKBH5* upregulation by rs151198415 C>CCACG change with a borderline significance (Figure 2B).

### 2.3 Survival Association, Validation, and Cis‐Regulatory Annotation for rs151198415 Locus

Survival analysis revealed that the rs151198415 CCACG insertion was associated with prolonged survival in 1523 NSCLC patients (Figure 2C,D) by Kaplan–Meier curve as well as a protective effect on death by multivariable Cox regression with adjustment for age, sex, smoking status, TNM stage and treatment (HR = 0.74, 95% CI = 0.58–0.95, *P* = 0.019, Table S12, Supporting Information). In subgroup analysis, this survival association of rs151198415 was more prominent in female patients and non‐smokers. A significant survival association was also observed in patients aged ≤ 60, those with stage I‐II disease, poor differentiation disease, and those who underwent surgery or did not receive target therapy (Figure S3A, Supporting Information). In the prospective SSACB cohort (Table S13, Supporting Information), we also observed a consistent trend toward a protective effect on death for rs151198415 (HR = 0.57, 95% CI = 0.26–1.28, *P* = 0.173) in incident lung cancer cases. Pooled meta‐analysis further confirmed this survival association (HR = 0.72, 95%CI = 0.57–0.92, *P* = 0.007, Figure S3B, Supporting Information). Based on the upregulation of *ALKBH5* by the rs151198415 variant, we then investigated the impact of *ALKBH5* expression on survival outcomes in other clinical cohorts. We used the Kaplan–Meier Plotter database and found a significant association between higher expression of *ALKBH5* and prolonged survival, as supported by two external NSCLC cohorts. (*n =* 181 in GSE50081 cohort and *n =* 226 in GSE31210 cohort) (Figure S4, Supporting Information.)

In A549 cells, IMR90 cells, and lung tissues, at the rs151198415 locus, we observed high enrichment of enhancer histone markers including H3K27ac and H3K4me1, but relatively less annotation of promoter marker H3K4me3 based on ChIP‐sequencing data from the Haploreg database (Figure 2E). In the assessment of chromatin accessibility, DNase signaling was detected by DNase sequencing in both the Haploreg (Figure 2E) and Regulome DB database (Figure 2G). Results of ATAC sequencing in Regulome DB also demonstrated the chromatin accessibility at the rs151198415 locus (Figure 2G). Interestingly, based on the organ‐specific functional annotation of the rs151198415 locus in the Regulome DB database, this locus was annotated as an enhancer in the lung (Figure 2F). As presented in Figure 2H using ENCODE ChIP‐sequencing data of 7 merged cell lines (GM12878, H1‐hESC, HSMM, HUVEC, K562, NHEK, and NHLF, Figure 2H), histone marker enrichment was also high for H3K27ac, H3K4me1 but was relatively low for H3K4me3 at the rs151198415 locus, with noteworthy DNase enrichment, further indicating the potential cis‐regulatory effect of rs151198415 locus on gene expression.

### 2.4 A Long‐Range Interaction of rs151198415 Locus with *ALKBH5* Promoter and EGR1‐Dependent Regulation of *ALKBH5* by rs151198415 Variant

**Figure 3:**
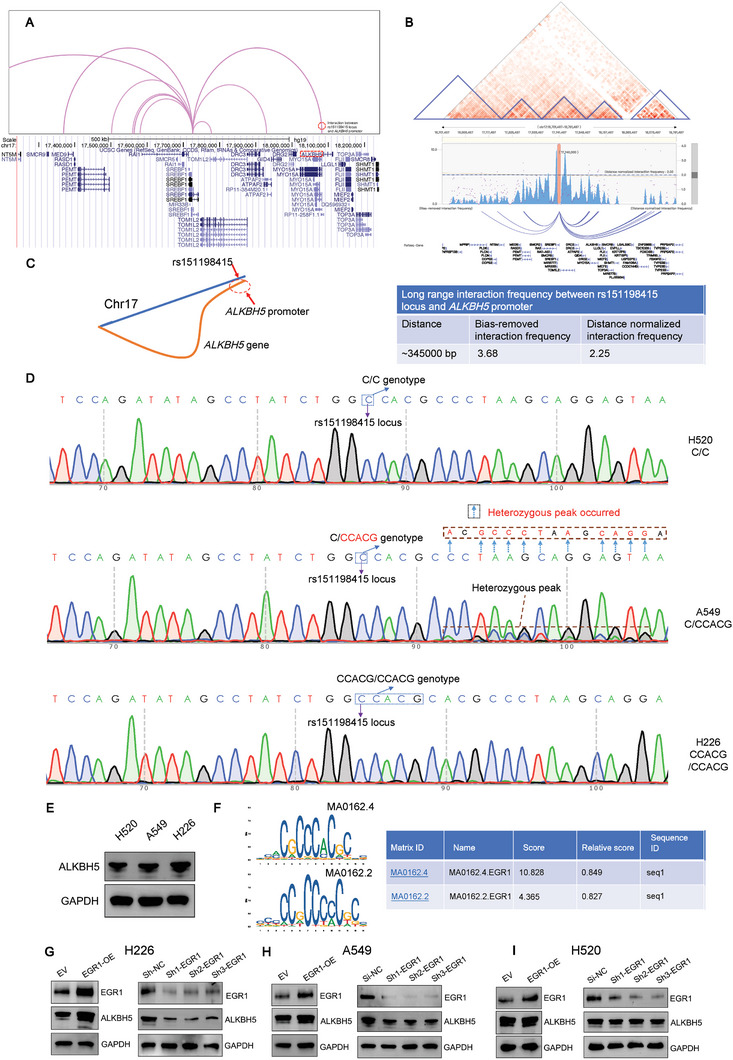
A) Long‐range interaction between the rs151198415 locus and *ALKBH5* promoter and EGR1‐dependent regulation of ALKBH5 by the rs151198415 variant. Capture‐HiC data from 3D Genome Browser indicated a notable long‐range interaction between the rs151198415 locus and *ALKBH5* promoter in lung tissues; B) Consistently, common Hi‐C data from 3DIV database demonstrated a long‐range interaction in A549 cells between rs151198415 locus and *ALKBH5* promoter, with a remarkable normalized interaction frequency; C) A schematic illustration depicts this potential long‐range interaction underlying the *ALKBH5* regulation by the rs151198415 variant; D) Sanger sequencing results for rs151198415 variant in H520, A549 and H226 cells, with rs151198415 locus, genotyping results, and heterozygous peak region (A549 cells only) marked; E) Western blot experiment in the three NSCLC lines revealed highest *ALKBH5* expression in H226 cells homozygous for the rs151198415 CCACG allele, further indicating *ALKBH5* upregulation by rs151198415 C>CCACG insertion; F) The rs151198415 C>CCACG change may create an EGR1 binding, as predicted by JASPAR tool. We presented the EGR1 binding motif and the binding score in the presence of rs151198415 CCACG allele; G) the Western blot experiment indicated positive regulation of ALKBH5 expression by EGR1 in H226 cells homozygous for the rs151198415 CCACG allele, H) however, this regulation was attenuated in heterozygous A549 cells, and I) nearly absent in H520 cells with common CC homozygous genotype.

Hi‐C and Capture Hi‐C data from 3DIV and 3D genome databases were examined to reveal the chromatin spatial interaction of the rs151198415 locus. Based on the eQTL evidence of *ALKBH5* upregulation by rs151198415 (Figure 2B), we found that the locus had a remarkable long‐range interaction with *ALKBH5* promoter based on the Capture Hi‐C evidence in lung tissue from the 3D genome browser (**Figure** **3**A). Consistent with Hi‐C results in A549 cells from the 3DIV database, the interaction between the rs151198415 locus and the *ALKBH5* promoter also seemed prominent (DNIF = 2.25, Figure 3B). Based on these results, we, therefore, plotted a spatial diagram for this long‐range interaction between the rs151198415 locus and *ALKBH5* promoter (Figure 3C). Collectively, these results indicated that rs151198415 locus might interact with the *ALKBH5* promoter to exert cis‐regulatory function. We next used Sanger sequencing and revealed C/C, C/CCACG, and CCACG/CCACG genotypes for rs151198415 in H520, A549, and H226 NSCLC cells, respectively (Figure 3D). In these 3 cell lines, we observed the highest ALKBH5 expression levels in H226 cells homozygous for the rs151198415 CCACG allele, further indicating the potential upregulation of ALKBH5 expression by rs151198415 C>CCACG change (Figure 3E).

As predicted by the JASPAR database, rs151198415 C>CCACG insertion might create a binding site for EGR1, with a relative prediction score of higher than 0.8 for CCACG allele (Figure 3F), but lower than 0.8 for C allele (Not shown). Importantly, we observed that EGR1 overexpression and knockdown, respectively, upregulated and downregulated *ALKBH5* expression in H226 cells, which was homozygous for the rs151198415 CCACG allele (Figure 3G). However, this regulation was impaired in A549 cells with a heterozygous genotype (Figure 3H), and even nearly vanished in H520 cells which were CC homozygous for rs151198415 (Figure 3I). These results implied the possible allelic difference in *EGR1* binding at rs151198415 locus to regulate *ALKBH5* expression.

### 2.5 Allele‐Specific Effect of rs151198415 Variant on EGR1 Binding to Regulate *ALKBH5* Transcription

**Figure 4:**
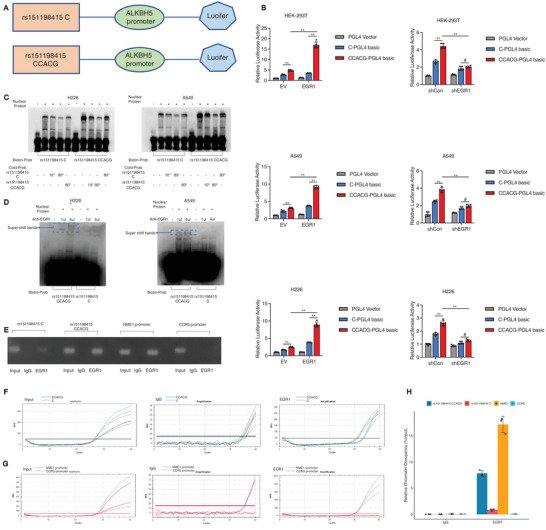
A) Allele‐specific effect of the rs151198415 variant on EGR1 binding to regulate *ALKBH5* transcription. Regions centered on the rs151198415 locus were cloned upstream of the *ALKBH5* promoter to construct a luciferase reporter system; B) In HEK‐293 T cells, *EGR1* overexpression promoted luciferase transcription driven by *ALKBH5* promoter and rs151198415 CCACG allele, while this promotion was attenuated in the presence of the C allele. Notably, *EGR1* downregulation inhibited the transcription more significantly with the CCACG allele compared to the C allele. Similar results were observed in A549 and H226 cells; C) In EMSA assay, increased protein binding was detected with the rs151198415 CCACG probe compared to the C probe in H226 and A549 cells. The binding to the biotin‐labeled probe could be completed by the corresponding cold probe; D) Adding the EGR1 antibody into the EMSA system led to a dose‐dependent supershift band in the presence of rs151198415 CCACG, which was attenuated with the C probe; E) ChIP assay was performed in A549 cells heterozygous for rs151198415, and in comparison with the C allele, gel electrophoresis demonstrated a higher level of rs151198415 CCACG allele in the DNA immunoprecipitated by EGR1 antibody. We also detected the NME1 promoter as the positive control, while the negative control CCR5 promoter was undetectable in the PCR products; F) In the representative Taqman ChIP‐qPCR, comparable amplification was observed between the C and CCACG allele in the A549 input group, however, amplification was greater for CCACG in comparison with the C allele in the EGR1‐immunoprecipitated group; G) Taqman ChIP‐qPCR also indicated substantial amplification for *NME1* promoter but not for *CCR5* promoter. Further quantitative analysis demonstrated preferred binding to the rs151198415 CCACG allele than C allele by EGR1, as higher levels of the CCACG allele were detected in the EGR1‐immunoprecipitated DNA. H) Quantification also confirmed positive EGR1 binding to the *NME1* promoter and lack of binding to the *CCR5* promoter. [Correction added on 20 December 2024 after online publication: Figure 4F and G are updated.]

To further study the regulatory effect of rs151198415 variant on *ALKBH5* transcription, we constructed PGL‐4.10 luciferase reporter vectors containing different alleles of rs151198415 and flanking sequences upstream of the *ALKBH5* promoter (**Figure** **4**A). In HEK‐293T cells, we found that the rs151198415 CCACG allele enhanced the transcriptional activity driven by *ALKBH5* promoter when compared with the C allele. Moreover, overexpression of *EGR1* promoted the transcription activity, magnifying the difference between CCACG and C alleles. On the contrary, EGR1 knockdown inhibited the overall transcription activity and impaired the contrast transcription activity in the comparison of rs151198415 CCACG vs. C allele (Figure 4B). Similar results were observed in A549 and H226 cells (Figure 4B). In A549 and H226 NSCLC cells, EMSA assay revealed stronger nuclear protein binding to the biotin‐labeled rs151198415 CCACG probe than the C probe (Figure 4C). The binding could be competed by the corresponding cold probe for both alleles, but the CCACG allele‐protein binding was harder to compete than the C allele (Figure 4C). The supershift band induced by the EGR1 antibody revealed stronger binding of EGR1 to the rs151198415 CCACG allele than the C allele in NSCLC cells (Figure 4D). Further ChIP experiment in heterozygous A549 cells showed that the DNA immunoprecipitated by EGR1 antibody contained more rs151198415 CCACG than C fragment, as indicated by agarose gel electrophoresis (Figure 4E), and Taqman qPCR (Figure 4F,H; Table S5, Supporting Information), supporting preferential binding of EGR1 to CCACG allele. According to the ChIP‐Atlas database, we also introduced NME1 and CCR5 promoters into the ChIP system as the positive and negative controls for binding to EGR1, respectively. The results showed substantial binding of EGR1 to *NME1* promoter, but not to *CCR5* promoter (Figure 4E,G,H; Table S5, Supporting Information), supporting the adequate quality control steps in the ChIP experiment.

### 2.6 The Impact of ALKBH5 on NSCLC Malignancy

**Figure 5:**
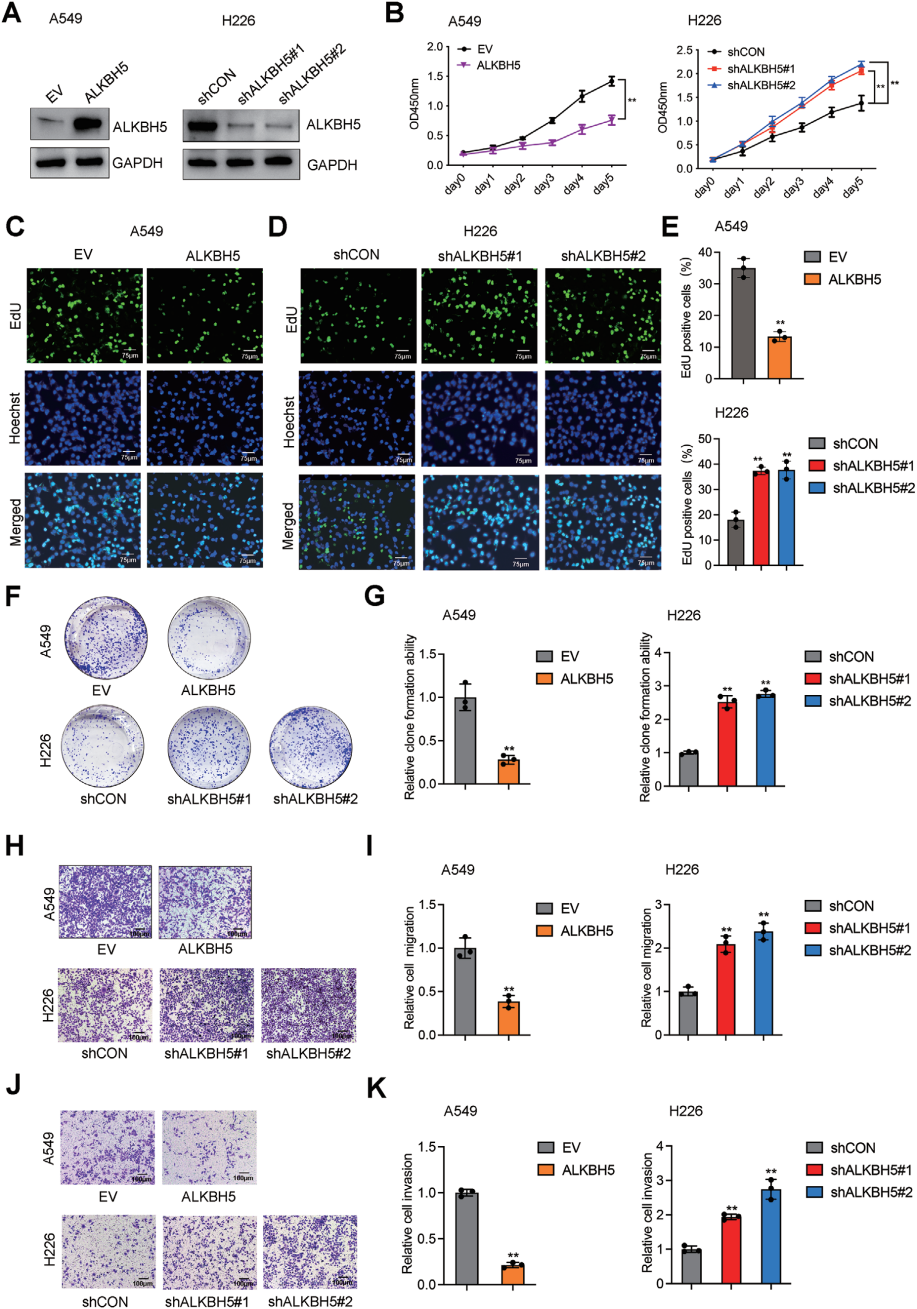
A) ALKBH5 inhibited malignancy of NSCLC cells. Western blot validated ALKBH5 overexpression in A549 and its knockdown in H226 cells; B) ALKBH5 overexpression inhibited the proliferation of NSCLC cells, and the knockdown of ALKBH5 promoted the proliferation of NSCLC cells, as indicated by CCK8 assay, C–E) EdU assay, and F–G) colony formation assay; H,I) Migration and J,K) invasion of NSCLC cells were also inhibited by *ALKBH5* overexpression but were promoted by its knockdown.

We next explored whether ALKBH5 could influence the malignant phenotype of NSCLC cells. ALKBH5 overexpression and knockdown were validated by western blot in A549 and H226 cells (**Figure** **5**A). We observed reduced cell viability with ALKBH5 overexpression based on CCK8 assay, and increased cell viability by ALKBH5 downregulation (Figure 5B). Similarly, results from EdU assay indicated that ALKBH5 overexpression inhibited cell proliferation (Figure 5C,E), while knockdown of the gene promoted proliferation (Figure 5D,E). As shown in the colony formation assay, ALKBH5 overexpression also suppressed cell proliferation, and its downregulation promoted cell proliferation (Figure 5F,G). Consistently, we observed impaired and restored migration ability induced by ALKBH5 overexpression and knockdown, respectively, as indicated by transwell assay without matrigel (Figure 5H,I). Similar trends were also observed for the invasiveness of NSCLC cells by transwell assay with matrigel (Figure 5J,K).

### 2.7 ALKBH5 Upregulated FBXL5 Via m^6^A Demethylation

**Figure 6:**
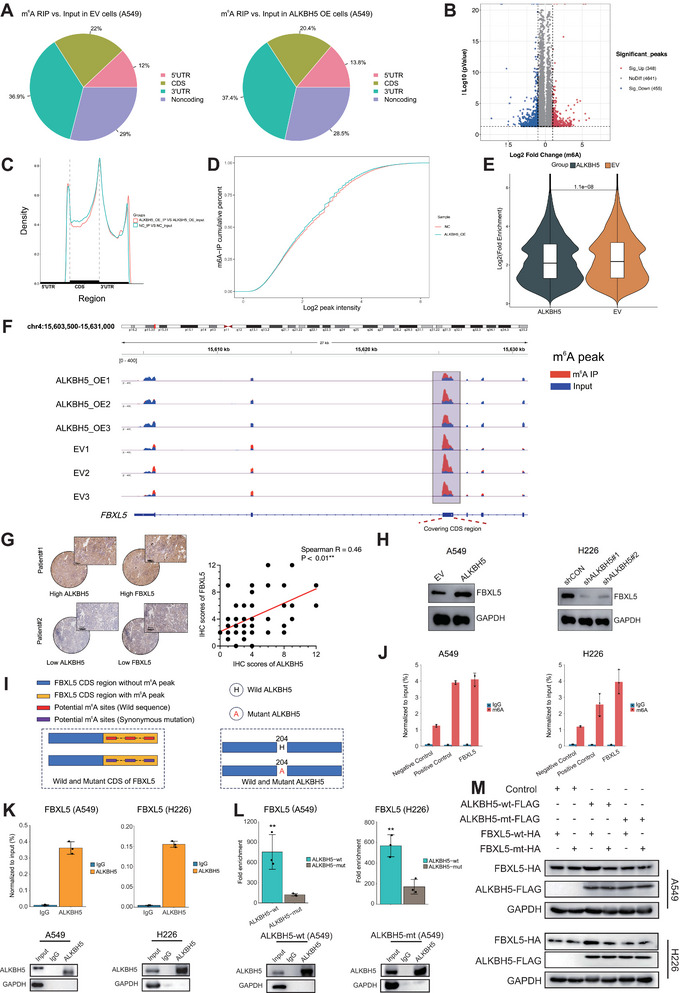
A)ALKBH5 upregulated FBXL5 expression by m^6^A demethylation. We performed m^6^A sequencing in A549 cells and identified m^6^A peaks across different regions in negative control (EV) and ALKBH5 overexpressed (ALKBH5 OE) cells; B) Volcano plot displayed significant m^6^A methylation peaks that were upregulated and downregulated by ALKBH5; C) We observed decreased m^6^A methylation (m^6^A demethylation) in the genomic CDS region; D) Cumulative percent and E) quantified changes for methylation indicated overall decreased methylation by ALKBH5 overexpression; F) Specific decreased m^6^A peak of the CDS region in *FBXL5* mRNA in the presence of ALKBH5 overexpression in A549 cells; G) IHC staining of NSCLC tissue microarray indicated a positive correlation between ALKBH5 and FBXL5 protein expression; H) Western blot revealed upregulated FBXL5 by ALKBH5 overexpression in A549 and H226 NSCLC cells; I) Graphical interpretation of *FBXL5* and *ALKBH5* mutations stably transfected into NSCLC cells; J) m^6^A‐RIP‐qPCR demonstrated m^6^A methylation in *FBXL5* mRNA; K) Common RIP‐qPCR demonstrated binding of ALKBH5 to *FBXL5* mRNA. Western blot was presented for quality control in protein extracted from the input group and the groups immunoprecipitated by IgG and ALKBH5 antibody; L) ALKBH5 H204A mutation impaired the binding of ALKBH5 to *FBXL5* mRNA as indicated by RIP‐qPCR. Western blot was performed for quality control using protein extracted from the corresponding groups; M) Western blot in NSCLC cells indicated that wild‐type ALKBH5 could upregulate wild‐type FBXL5, but this regulation was attenuated in the presence of ALKBH5 H204A mutation or FBLX5 synonymous mutation in potentially m^6^A sites.

We performed MeRIP‐seq and RNA sequencing to investigate ALKBH5‐mediated m^6^A methylation and found that the m^6^A methylation was affected by ALKBH5 overexpression across different genomic regions (**Figure** **6**A). The m^6^A methylation peaks were altered in multiple genes, including 348 significant upregulations and 455 downregulations (Figure 6B). Although ALKBH5 overexpression alleviated m^6^A methylation on an overall genomic scale (Figure 6D,E), decreased m^6^A methylation seemed most prevalent in the CDS regions (Figure 6C). We analyzed the m^6^A and RNA sequencing data in combination and found that *FBXL5* was one of the top genes with significantly decreased m^6^A methylation and increased mRNA expression by ALKBH5 overexpression in A549 cells (Table S14, Supporting Information). As visualized in Figure 6F, we observed obvious FBXL5 m^6^A demethylation in its mRNA CDS region induced by ALKBH5 overexpression. According to IHC staining in NSCLC tissue microarray, a significant positive correlation was found between ALKBH5 and FBXL5 protein expression (Figure 6G). ALKBH5 overexpression in A549 cells upregulated FBXL5, and the knockdown of ALKBH5 downregulated FBXL5, as observed in H226 cells (Figure 6H). As presented in Figure 6I, we next extracted the m^6^A peaks region in *FBXL5* mRNA from m^6^A sequencing data. We predicted the potential m^6^A sites by the SRAMP database to design synonymous mutations, which was presented in Table S7 (Supporting Information) in detail. ALKBH5 H204A mutation was also designed for further experiments (Figure 6I). Further, m^6^A‐RIP‐qPCR validated the FBXL5 m^6^A methylation in NSCLC cells (Figure 6J). Moreover, RIP‐qPCR confirmed the binding of ALKBH5 to *FBXL5* mRNA (Figure 6K), but the binding affinity was impaired in the presence of ALKBH5 H204A mutation (Figure 6L, Figure S5, Supporting Information). Interestingly, in the presence of ALKBH5 H204A mutation or *FBXL5* synonymous mutation at the potential m^6^A sites, FBXL5 was unable to be upregulated by ALKBH5, suggesting the ALKBH5 protein and FBXL5 m^6^A sites were both responsible for m^6^A modification in NSCLC cells (Figure 6M).

### 2.8 The ALKBH5‐FBXL5 Axis Plays a Tumor Suppressive Role Via Influencing Intracellular ROS and the PI3K‐AKT and NF‐κB Pathways

**Figure 7:**
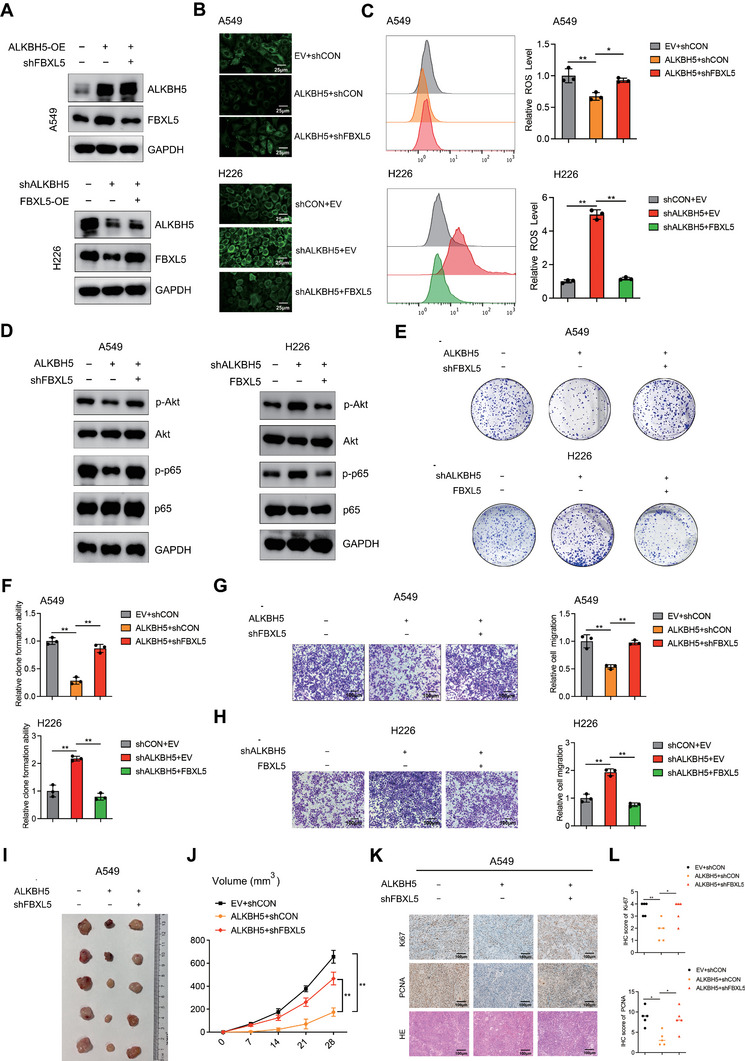
A)ALKBH5‐FBXL5 axis decreased intracellular ROS and inhibited PI3K‐AKT and NF‐κB pathway activations to exert a cancer suppressor role in NSCLC. ALKBH5 overexpression and FBXL5 knockdown were performed in A549 cells and the opposite manipulation was performed in H226 cells, which was validated by western blot; B) ROS fluorescent probe staining and C) flow cytometric detection indicated decreased ROS levels by ALKBH5 overexpression, which was retrieved by FBXL5 knockdown in A549 cells, and opposite results were observed by in H226 cells with the corresponding manipulation; D) p‐AKT and p65, two key elements for PI3K and NF‐κB pathways activation, were downregulated by ALKBH5 overexpression, which could be reversed by FBXL5 downregulation in A549 cells. Conversely, PI3K and NF‐κB pathways could be activated by ALKBH5 downregulation, which was attenuated by FBXL5 upregulation in H226 cells; E,F) Colony formation and G,H) migration of A549 cells were inhibited by ALKBH5 overexpression but were retrieved by FBXL5 downregulation, and vice versa for the impact of ALKBH5‐FBXL5 axis on colony formation and migration in H226 cells I,J) Similarly, in vivo experiments demonstrated that FBXL5 was also indispensable for ALKBH5‐induced shrinkage of xenograft tumors constructed by A549 cells; K,L) IHC experiments of xenograft tumors for Ki‐67, PCNA, indicated less staining by ALKBH5 overexpression, which was partly restored in the presence of additional FBXL5 knockdown. HE was used as a background staining marker for NSCLC cells.

**Figure 8:**
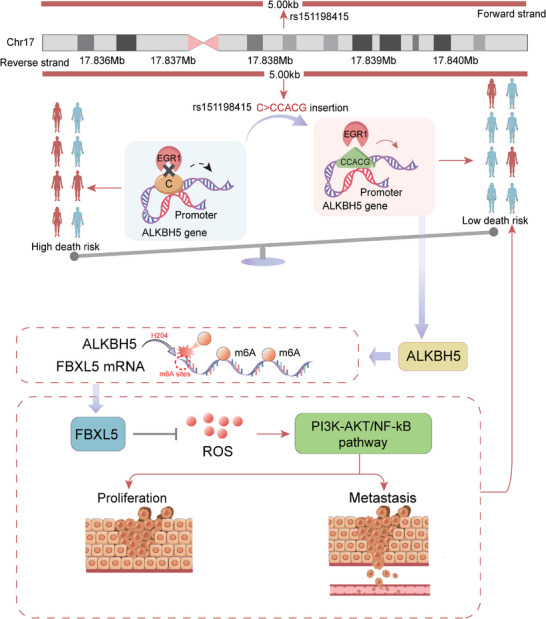
Schematic model for the molecular mechanism underlying the association of rs151198415 variant with the survival of NSCLC patients in the present study.

FBXL5 has been identified as a downstream effector of ALKBH5, whose ablation was previously reported to alleviate iron overload in cancer cells, leading to a rise in oxidative stress and, as a result, compensatory proliferation in cancer cells.^[^ ^16^ ^]^ Therefore, conversely, we hypothesized that ALKBH5‐FBXL5 exerted tumor suppressive function via ROS reduction. To validate this functional dependence on the ALKBH5‐FBXL5 axis, we implemented overexpression of ALKBH5 and knockdown of FBXL5 in A549, along with opposite manipulation in H226 cells (**Figure** **7**A). As indicated by ROS fluorescent probe staining (Figure 7B) and flow cytometry quantification (Figure 7C), ALKBH5 overexpression led to a reduction in ROS levels, which was restored by FBXL5 knockdown in A549 cells. On the other hand, ALKBH5 downregulation elevated ROS levels in H226 cells, but this ROS elevation was impeded by FBXL5 upregulation (Figure 7B,C). Previous studies revealed PI3K‐AKT and NF‐κB pathway activation were induced by intracellular ROS,^[^ ^17^ ^]^ which urges us to test the involvement of the two oncogenic pathways in the ALKBH5‐FBXL5 axis. In A549 and H226 cells, gain‐ and loss‐of‐function studies demonstrated that the inhibited phosphorylation of p65 and AKT by ALKBH5 overexpression was reversed by FBXL5 knockdown, and the promoted phosphorylation by ALKBH5 knockdown was impeded by FBXL5 overexpression (Figure 7D). We next investigated whether the impact of the ALKBH5‐FBXL5 axis on the oncogenic pathways could impair the malignancy of NSCLC cells. In A549 cells, the proliferation (Figure 7E,F; Figure S6A,C, Supporting Information) and migration (Figure 7G) of cells were inhibited by ALKBH5 overexpression, an effect that was counteracted by simultaneous FBXL5 knockdown. Conversely, the opposite effect was observed in H226 cells (Figure 7E,F,H; Figure S6B,D, Supporting Information). Similar results were observed for the invasiveness of NSCLC cells (Figure S6E–H, Supporting Information). In vivo results also demonstrated tumor xenografts shrinkage upon ALKBH5 overexpression (Figure 7I,J), but this effect was weakened when FBXL5 was knocked down. The ALKBH5‐FBXL5 axis also inhibited Ki‐67 and PNCA protein expressions in the tumor xenografts, as indicated by IHC staining (Figure 7K,L), indicating the suppressed proliferation by this axis in vivo. Finally, we summarized the survival association of the rs151198415 variant in NSCLC patients and elucidated the underlying mechanism in a schematic plot (**Figure** **8**).

## 3 Discussion

Exploring the association of genetic variants with patients’ survival holds promise for advancing our understanding of individualized survival surveillance in cancer, a focus of our recent research endeavors. Given the wide recognition of m^6^A modification as an essential RNA modification in cancer, it is conceivable that it may regulate multiple genes, including those we have previously investigated.^[^ ^18^, ^19^ ^]^ However, the interaction between the m^6^A pathway and genetic variants in NSCLC remains unclear. In the present study, we found that genetic variants with the potential to regulate m^6^A pathway might also modulate the survival of NSCLC patients. Specifically, the rs151198415 C>CCACG insertion may enhance the binding of EGR1, thereby remotely upregulating *ALKBH5* expression, which in turn leads to upregulation of *FBXL5* expression via m^6^A demethylation. Ultimately, the ALKBH5‐FBXL5 axis reduced intracellular ROS levels, thereby inhibiting the activation of PI3K‐AKT and NF‐κB pathways. This cascade of events culminates in impaired cancer malignancy and supports the impact of the rs151198415 insertion on the prolonged survival of NSCLC patients.

Notably, ALKBH5 has been implicated in a series of studies as a tumor suppressor in NSCLC,^[^ ^20^, ^21^, ^22^, ^23^ ^]^ and our results in the present study were in line with these findings. For NSCLC, m^6^A modification was reported to exert influence not only on cancer progression, but also metabolism,^[^ ^24^ ^]^ and response to immunotherapy.^[^ ^25^ ^]^ For example, the IGF2BP3‐COX6B2 axis has been identified to be regulated in an m^6^A‐dependent manner in NSCLC, contributing to alterations in nicotinamide metabolism and resistance to EGFR‐TKI therapy.^[^ ^46^ ^]^ Furthermore, METTL3/YTHDF2‐mediated m^6^A modification of the downstream genes has been correlated with PDL1 expression and CD8+ T cell infiltration in NSCLC, suggesting potential avenues for enhancing anti‐PDL1 immunotherapy.^[^ ^25^ ^]^ Therefore, the role of m^6^A pathway is crucial in NSCLC progression and treatment response. In the present study, we observed upregulated FBXL5 expression resulting from ALKBH5‐induced m^6^A demethylation and delineated the role of the ALKBH5‐FBXL5 axis in impeding the growth and metastasis of NSCLC cells. FBXL5, also previously identified as an onco‐suppressor,^[^ ^16^, ^26^, ^27^ ^]^ exhibits an adverse association with the survival of cancer patients.^[^ ^28^ ^]^ Downregulation of FBXL5 has been shown to elevate intracellular ROS levels via iron overload, although it was unable to induce ferroptosis.^[^ ^16^ ^]^ Our observations in NSCLC cells further underscore the dependency of ALKBH5 on FBXL5 to decrease intracellular ROS, thereby inhibiting oncogenic pathways and malignant phenotype. Moreover, we identified the ALKBH5 H204 region critical for interacting with specified m^6^A sites in FBXL5 mRNA to facilitate the m^6^A modification, highlighting the importance of protein and RNA structure in orchestrating the ALKBH5‐FBXL5 axis.

Previous studies have primarily focused on elucidating the m^6^A modification mechanisms underlying the regulation of downstream genes and subsequent phenotypic differences in NSCLC.^[^ ^12^, ^13^, ^14^, ^24^, ^25^, ^29^ ^]^ In contrast, the present study delves into the functional roles of genetic variants in the upstream regulation of an m^6^A pathway gene. Enhancer–Promoter (E–P) interaction is a critical mechanism underlying the mechanism of observed phenotypes induced by genetic variants. For example, previous studies implied that genetic variants at 16p13 interact remotely with the promoter of lncRNA *GCLET*, conferring the risk of gastric cancer.^[^ ^30^ ^]^ Similarly, risk loci for colorectal cancer^[^ ^31^ ^]^ and esophageal squamous cell carcinoma,^[^ ^32^ ^]^ reportedly regulate the expression *CATSPERE* and *PLCE1*, through long‐range interactions with their promoters and by altering TF binding. Our laboratory also previously demonstrated the remote E–P interactions between genetic variants and gene promoters, providing a mechanistic basis for the observed survival associations of genetic variants in cancer patients.^[^ ^8^, ^9^ ^]^ In the present study, we first identified the rs15118415 locus within a putative enhancer region that highly interacts with the *ALKBH5* promoter. Through this remote interaction, we found that the rs151198415 C>CCACG insertion engages with EGR1 to regulate *ALKBH5* expression. This novel E‐P interaction supports the biological relevance of the survival association observed for the rs151198415 variant. However, beyond rs151198415, other genetic variants may also regulate *ALKBH5* expression, which will be investigated in our future studies.

The EGR1‐ALKBH5 regulatory axis was also one of the important findings. EGR1 is a well‐known TF that regulates the transcription of multiple genes or microRNAs, including *KRT18*,^[^ ^33^ ^]^ *PDK1*,^[^ ^34^ ^]^ and miR‐139^[^ ^35^ ^]^ in NSCLC cells. To our knowledge, no studies have reported the regulation of *EGR1* on *ALKBH5* in cancer. Another important finding in the present study was that EGR1 regulates *ALKBH5* in an rs151198415 allele‐dependent manner, likely due to the impact of this genetic variant on EGR1 binding affinity. These findings further underscore the important role of genetic variants in influencing the TF‐gene regulatory network.

Some limitations in the present study warrant consideration. First, the survival association for the rs151198415 variant was identified in a retrospective study and a small prospective study, future prospective population studies are required for validation. Second, certain potential eQTL variants for *ALKBH5*, as suggested by GTEx, were not included in the present study due to their relatively low imputation quality. Last, as the present study focuses on common germline variants, it is important to investigate additional variants with minor allele frequency <5% in larger population studies to assess their impact comprehensively.

In conclusion, our study highlights a noteworthy survival association in NSCLC patients for the genetic variants capable of regulating the m^6^A eraser, *ALKBH5*. Specifically, the rs151198415 locus exerts remote interaction with the *ALKBH5* promoter, and the C>CCACG insertion enhances EGR1 binding to upregulate *ALKBH5* expression. ALKBH5, in turn, upregulates *FBXL5* through m^6^A demethylation, with this regulatory process dependent on the interaction between specified H204 sites in the ALKBH5 protein and potential m^6^A sites in *FBXL5* mRNA. Moreover, the ALKBH5‐FBXL5 axis reduced ROS levels, inhibited the activation of the PI3K‐AKT and NF‐κB pathway, and ultimately impaired NSCLC malignancy. This molecular mechanism likely underlies the association of rs151198415 C>CCACG change with prolonged survival in NSCLC patients.

## 4 Experimental Section

### Study Population, Genetic Variants, and Survival Association

The discovery dataset included 1523 NSCLC patients from the ongoing clinical genome‐wide association study (GWAS) described in a previous study.^[^ ^8^ ^]^ Whole blood DNA was extracted from the NSCLC patients and genotyping was performed with Illumina Infinium Global Screening Assay (GSA, GSAMD‐24v1‐0, Illumina, US). Genotyping results were then subjected to imputation using IMPUTE2 (version 2.3.2) software with genomic data of Eastern Asians from the 1000 Genomes project as a reference. In the present study, 30 m^6^A pathway genes were included, including 2 erasers, 13 readers, and 10 writers, along with 5 epigenetic regulator genes related to m^6^A modification (Table S1, Supporting Information). Among the 30 genes, 23 were selected from one previous study^[^ ^36^ ^]^ that focused on the m^6^A pathway genetic variants and cancer etiology [*WTAP, METTL3, METTL14, METTL16, RBM15, RBM15B, VIRMA* (also termed *KIAA1429*), *ZC3H13, FTO, ALKBH5, YTHDF1, YTHDF2, YTHDF3, YTHDC1, YTHDC2, EIF3A, EIF4E, HNRNPC, HNRNPA2B1, IGF2BP1, IGF2BP2, IGF2BP3, and LRPPRC*], other included genes include a well‐recognized m^6^A writer (*METTL4*)^[^ ^37^ ^]^ and reader (*SND1*),^[^ ^38^ ^]^ and 5 genes that interacted with m^6^A modification (*ADAR, ADARB1, DGCR8, DICER1*, and *DROSHA*).^[^ ^39^ ^]^ Using GTEx database,^[^ ^40^ ^]^ SNPs were identified that were significant expression quantitative trait loci (eQTL) of the 30 genes. The identified SNPs were extracted from the imputed GWAS dataset and subjected to quality control. The included SNPs fulfilled the following criteria: imputation quality (*r* ^2^ > 0.8), minor allele frequency (>5%), call rate (>95%), and no deviation from Hardy‐Weinberg equilibrium (*p* > 0.05). A Multivariable Cox proportional hazards model was adopted for survival analysis. False probability report probability (FPRP)^[^ ^41^ ^]^ and Bayesian false‐discovery probability (BFDP)^[^ ^42^ ^]^ tests were performed as statistical corrections to control for false positive results in multiple testing analyses. Briefly, the FPRP calculation was based on the prior probability (0.1), prior hazard ratio (HR) (1.5), and the actual HRs. BFDP was a Bayesian‐based statistical method considering the minor allele frequency and the survival association of SNPs. FPRP and BFDP were controlled at levels as low as 0.2 and 0.8, respectively, to avoid false positive findings. Associations were considered truly significant only if the following criteria were met (*p* < 0.05, FPRP<0.2, and BFDP<0.8). After computing the linkage disequilibrium (LD) among the target SNPs by PLINK software (version 1.90, US), tag SNPs were further selected for survival prediction modeling in combination.

### Functional Variant Prediction, eQTL Validation, and Survival Association Validation

Survival‐associated SNPs were then subjected to functional prediction using the Regulome DB database.^[^ ^43^ ^]^ Only genetic variants with evidence of transcriptional factor (TF) binding motifs and DNase footprints (Regulome DB ranking score 1a and 1b) were selected. The eQTL effect of the potentially functional variants was further validated in 445 lymphoblastoid cells from the 1000 Genomes (1000G) Project (Geuvadis, E‐GEUV‐1).^[^ ^44^, ^45^ ^]^

SNP‐gene correlation analysis was also performed for the eQTL‐validated variant using 74 NSCLC tissue biopsy samples collected from August 2024 to October 2024 at Shanghai Chest Hospital. DNA and RNA were extracted from the tissues, and the RNA was reverse‐transcribed into cDNA for real‐time quantitative PCR (qPCR) (Takara, Japan). Sanger sequencing was used to genotype the eQTL‐validated variant using the extracted DNA, with primers shown in Table S2 (Supporting Information). Finally, the eQTL was validated for their survival association using the prospective population cohort Shanghai Suburban Adult Cohort and Biobank (SSACB). Until December 2022, a total of 298 incident lung cancer cases occurred since June 2016, who were free from cancer at baseline. Blood samples of these patients collected from baseline were genotyped with the Infinium Chinese Genotyping Array‐24 v1.0 Beadchip (Illumina, US) and imputed by the Westlake BioBank Server (WBBC Phase 1+1000G Phase 3 as reference). Patients were excluded from survival analysis with unavailable information on diagnosis time (*n =* 13), history of operation (*n =* 42), or history of radio/chemotherapy (*n =* 6). A total of 237 eligible patients were included in the final survival validation. Written informed consent was obtained from all participants for research purposes. All patients signed informed consent for research purposes. The association of *ALKBH5* expression and the survival of NSCLC patients was also investigated using the Kaplan–Meier Plotter database,^[^ ^46^ ^]^ with the best cutoff for gene expression set as default. The study was reviewed and approved by the Medical Research Ethics Committee of the Shanghai Chest Hospital (IRB No.: KS24037), School of Public Health, Fudan University (IRB No.: 2016‐04‐0586), and Fudan University Shanghai Cancer Center (FUSCC, IRB No.: 050432‐4‐1805C).

### Cell Lines and Culture

For the functional investigation of the potentially functional SNP rs151198415, genotypes of the NSCLC cell lines were first determined by Sanger sequencing using specified primers (Table S2, Supporting Information). Finally, A549, NCI‐H226 (H226), and NCI‐H520 (H520) cell lines with different genotypes were selected for further experiments. Moreover, HEK‐293T cells were used as a tool in luciferase assay. These 4 cell lines were authenticated using the Short Tandem Repeat (STR) method. NSCLC cells overexpressed the gene coding sequence or shRNAs knockdown, respectively (Table S3, Supporting Information). The cell culture conditions were described in the previous study.^[^ ^9^ ^]^

### Cis‐Regulatory Annotation, Chromatin Spatial Interaction, Western Blot, Chromatin Immunoprecipitation‐qPCR, Electrophoresis Mobility Shift, and Dual‐Luciferase Reporter Assay

To identify the cis‐regulatory function of the DNA region covering the rs151198415 locus, the enrichment of histone markers, including H3K27ac, H3K4me1, and H3K4me3 by Haploreg^[^ ^47^ ^]^ and ENCODE^[^ ^48^, ^49^ ^]^ databases were first assessed. Further organ‐specific enhancer annotation and DNase‐based chromatin accessibility were visualized in the Regulome DB database. Chromatin accessibility was also presented by the Assay for Transposase‐Accessible Chromatin using sequencing (ATAC‐seq) data from the Regulome DB database. Hi‐C data from the 3DIV database^[^ ^50^, ^51^ ^]^ and Capture‐HiC data from the 3D genome database,^[^ ^52^ ^]^ respectively were investigated, to investigate the chromatin spatial interaction between the rs151198415 locus and *ALKBH5* promoter. For the 3DIV Hi‐C data, Distance Normalized Interaction Frequency (DNIF) was used to show the strength of spatial interaction between the two DNA regions (DNIF>2 was considered prominent long‐range interaction). JASPAR database^[^ ^53^, ^54^ ^]^ was used to predict the potential differential TF binding to rs151198415 locus in an allele‐specific manner. Western blot, electrophoretic mobility shift assay (EMSA), and chromatin immunoprecipitation (ChIP) experiments were carried out in NSCLC cells to validate the predicted DNA‐TF interaction. Briefly, as indicated in the previous study,^[^ ^9^ ^]^ proteins extracted from NSCLC cells were separated by SDS‐polyacrylamide gel electrophoresis, followed by transfer, antibody incubation, and imaging. EGR1 (Cell Signaling Technology, US), ALKBH5 (Proteintech, China), and GAPDH (Proteintech, China) antibodies were used as primary antibodies in western blot assay to test the impact of EGR1 on ALKBH5. For EMSA experiments, the nuclear protein of NSCLC cells was extracted and incubated with biotin‐labeled/competitor cold probes (Table S4, Supporting Information) and the reaction mix was prepared by EMSA kit (Thermo Fisher Scientific, US). After gel electrophoresis and blot transfer, the protein‐oligo complex can be detected by chemiluminescence. Unlabeled probes representing different alleles were used to compete for the binding of biotin‐labeled probes with nuclear proteins in an allele‐specific manner. The EGR1 antibody was used to perform supershift EMSA. The ChIP experiment was conducted with the following 4 steps: DNA‐protein crosslink, cell lysis, immunoprecipitation, and DNA purification and recovery. The EGR1 and IgG antibody was also used to precipitate the bound DNA during immunoprecipitation. Then, purified DNA was quantified by Taqman qPCR and visualized by agarose gel electrophoresis using specified primers (Table S5, Supporting Information). ChIP‐Atlas was a database that incorporated ChIP sequencing evidence from multiple cells/tissues.^[^ ^55^ ^]^ This database was used and selected the promoter of NME1 and CCR5, respectively, based on the binding score to EGR1. The NME1 promoter had the highest binding score, while the CCR5 promoter had a binding score of 0. Therefore, the NME1 and CCR5 promoters were selected to design primers as positive and negative controls for the quantification of purified DNA. A pGL4.10 luciferase reporter vector (Promega, US) was constructed by cloning the rs151198415‐flanking region (≈500bp) into the upstream of the *ALKBH5* promoter to reveal the effect of rs151198415 variant on *ALKBH5* transcriptional activity. The relative activity of Firefly and Renilla was detected by the Dual‐Luciferase Reporter Assay System (Promega, US).

### Methylated RNA Immunoprecipitation (MeRIP) Sequencing, MeRIP‐qPCR, and Mutational Vector Construction

A549 cells with ALKBH5 overexpression and the relative control were subjected to methylated RNA immunoprecipitation (MeRIP). Fragmented mRNA (100‐nt) was incubated with m^6^A polyclonal antibody and protein A/G magnetic beads to perform target RNA immunoprecipitation. Target RNAs bound to m^6^A antibodies were eluted and purified for m^6^A sequencing or MeRIP‐qPCR. MeRIP‐sequencing was performed at Lc‐Bio Technologies (Hangzhou, China). To detect the m^6^A modification of target RNA, MeRIP‐qPCR was performed in the lab using the riboMeRIPTM m^6^A Transcriptome Profiling Kit (RiboBio, China), according to the manufacturer's instructions. The kit contained negative control and positive control primers for quality control for qPCR analysis. The RNA of interest was immunoprecipitated, isolated, and quantified by specifically designed qPCR primers (Table S6, Supporting Information) based on the relative peak signaling from MeRIP‐sequencing. Common RNA immunoprecipitation (RIP) was also conducted using the EZ‐Magna RIP™ RNA‐Binding Protein Immunoprecipitation Kit (Sigma, US). Anti‐ALKBH5 antibody was used in RIP to precipitate the possible FBXL5 mRNA for qPCR using specific primers (Table S6, Supporting Information). FBXL5 antibody (Abcam, UK) was also purchased to validate the ALKBH5‐FBXL5 regulation axis. The potential m^6^A sites in FBXL5 mRNA were predicted by the SRAMP database,^[^ ^56^ ^]^ and then introduced the H204A mutation for ALKBH5 and synonymous mutations for putative m^6^A sites in FBXL5 (Table S7, Supporting Information), to construct FLAG‐tagged (ALKBH5‐MT‐FLAG) and N‐terminal HA‐tagged (FBXL5‐MT‐HA) lentiviral expression vectors, respectively, for stable transfection in NSCLC cells. Western blot was used to detect whether the impact of ALKBH5 on FBXL5 was dependent on potential m^6^A sites in the FBXL5 coding sequence (CDS) region and ALKBH5 H204 protein region, with FLAG and HA antibody (Proteintech, China).

### PI3K‐AKT and NF‐κB Pathways Activation, Colony Formation, EdU, Transwell, CCK8, Reactive Oxygen Species (ROS) Assay

Western blot was performed to test PI3K‐AKT and NF‐κB pathways activation with AKT/pAKT (Proteintech, China) and p65/p‐p65 (Cell Signaling Technology, US) antibodies, respectively. Cells were seeded into 96‐well plates at the density of 2000 cells per well for cell viability test using CCK8 assay (Gaithersburg, MD, US). The absorbance at 450nm was detected. Colony formation and transwell assay were described in the previous studies.^[^ ^7^, ^9^ ^]^ Cellular Reactive Oxygen Species (ROS) levels were quantified by ROS Assay Kit (Beyotime Biotechnology, China) via flow cytometry and fluorescent probe staining using a DCFH‐DA probe and relatively positive controls. 5‐ethynyl‐2’‐ deoxyuridine (EdU) and Hoechst 33342 dyes from related kits (RiboBio, China) were used to stain for cell proliferation and nuclei visualization in NSCLC cells. Cells were seeded at a density of 5 × 10^3^ cells per well in a 96‐well plate and stained for 4 h. After staining, cells were fixed and the staining was captured using laser scanning confocal microscopy (Leica, Germany).

### Tissue Microarray and Animal Models

To validate the relationship between FBXL5 and ALKBH5 expression in the clinical stage, NSCLC tissue microarrays were obtained from Shanghai Outdo Biotech Company that contained 120 tumor tissues from NSCLC patients (Microarray ID: HLugA120PG01), which was approved by the ethics committee of Shanghai Outdo Biotech Company (IRB No: SHYJS‐CP‐1510004). Based on the method of the previous publication,^[^ ^57^ ^]^ IHC staining for ALKBH5 and FBXL5 proteins were performed, and evaluated the staining score for analysis. Animal experiments were conducted in accordance with guidelines of the Committee on the Ethics of Animal Experiments of Shanghai Chest Hospital [IRB No: KS24037]. BALB/c nude mice weighing 18–20 g and aged 4–6 weeks were used to construct A549 xenograft tumors. Mice were subcutaneously injected with 2 × 10^6^ A549 cells in PBS solution. The mice were divided into 3 groups (*n =* 5 per group) based on the injected cells: the control group injected with empty vector cells; the group with cells stably overexpressing both *ALKBH5* CDS region and empty control vector group; and the group stably overexpressing both *ALKBH5* and *FBXL5* shRNA. Tumor size was measured weekly using a caliper under sterile feeding conditions, and tumor volume was calculated using the formula (length × width^2^)/2. In the 4th week, mice were euthanized, and tumors were dissected for IHC staining, including hematoxylin and eosin (HE), Ki‐67, and PNCA.

### Statistical Analysis

Survival analysis was performed using the Cox proportional hazards model and Kaplan–Meier analysis. BFDP and FPRP tests were applied in SNP‐based association analysis to control the false positive rate. Group differences were analyzed using a t‐test, ANOVA test, and rank‐sum test, depending on the data's normal distribution. Parametric or nonparametric tests for time‐series data were used in animal or CCK8 experiments, depending on the normal distribution of the data. Quantitative data among groups were presented as Mean ± SD and differences were considered significant at * *p* < 0.05; and ** *p* < 0.01.

### Ethics approval and patient consent statement

Patient tissue or blood samples were collected for research purposes with ethical approval, and all animal experiments were conducted in adherence to ethics committee guidelines. This study was approved by the ethics committees of Fudan University Shanghai Cancer Center (IRB No: 050432‐4‐1805C), the School of Public Health, Fudan University (IRB No: 2016‐04‐0586), Shanghai Outdo Biotech Company (IRB No: SHYJS‐CP‐1510004), and Shanghai Chest Hospital (IRB No: KS24037).

## Conflict of Interest

The authors declare no conflict of interest.

## Supporting information



Supporting Information

Supporting Information

## Data Availability

The data that support the findings of this study are available from the corresponding author upon reasonable request.
