# Supplementary material for: Cis‐Regulation of an m6A Eraser by an Insertion Variant Associated with Survival of Patients With Non‐Small Cell Lung Carcinoma
Source: Adv Sci (Weinh). 2024 Dec 16;12(5):2407652. doi: 10.1002/advs.202407652 (PMC11791940; doi:10.1002/advs.202407652)
Supplement: Supplementary file 1 — Supporting Information [file ADVS-12-2407652-s001.pdf]

ADVANCED SCIENCE
Open Access

Supporting Information

for *Adv. Sci.*, DOI 10.1002/advs.202407652

Cis-Regulation of an m^6A Eraser by an Insertion Variant Associated with Survival of Patients With Non-Small Cell Lung Carcinoma

Lei Cheng, Qiangsheng Hu, Yanan Wang, Wei Nie, Haijiao Lu, Bo Zhang, Genming Zhao, Shiyun Ding, Feng Pan, Yinchen Shen, Runbo Zhong and Ruoxin Zhang**

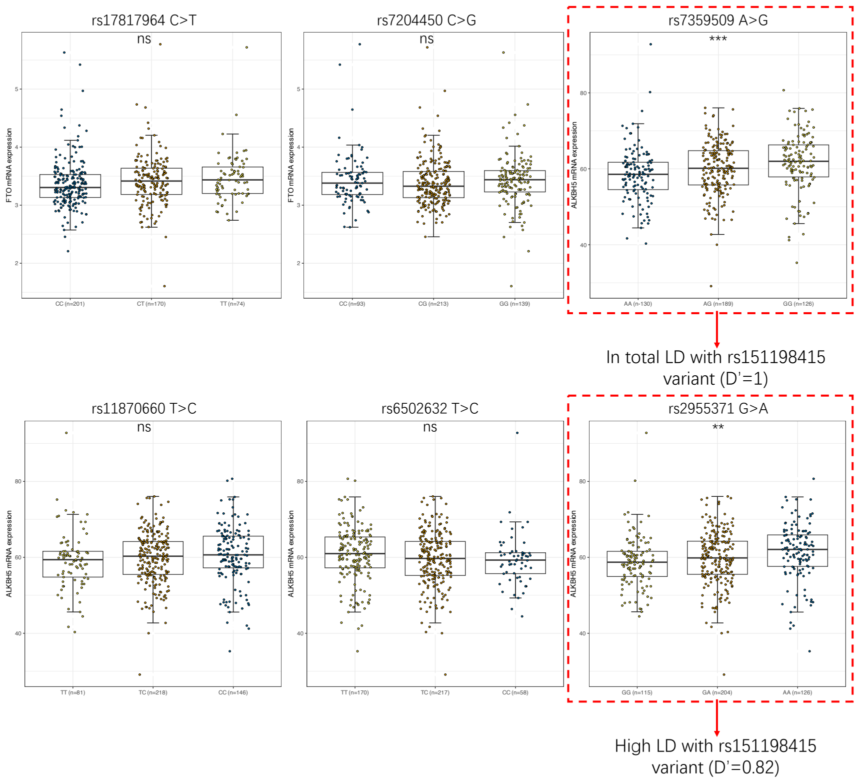

Figure S1: Genotype-phenotype validation of potentially functional variants using 1000 Genomes dataset.

Using transcriptome and genotype data from 445 lymphoblastoid cell lines in the 1000G database, we validated the genotype-phenotype association in genetic variants with the 7 top functional score predicted by Regulome DB database. Results for rs151198415 were presented in Figure 2B. This figure presented the validation results for the other 6 variants, including rs17817964, rs7204450, rs7359509, rs11870660, rs6502632, and rs2955371, among which rs7359509 A>G and rs2955371 G>A could significantly upregulate *ALKBH5* mRNA expression and were in high LD with rs151198415 variant.

Abbreviations: LD, linkage disequilibrium.

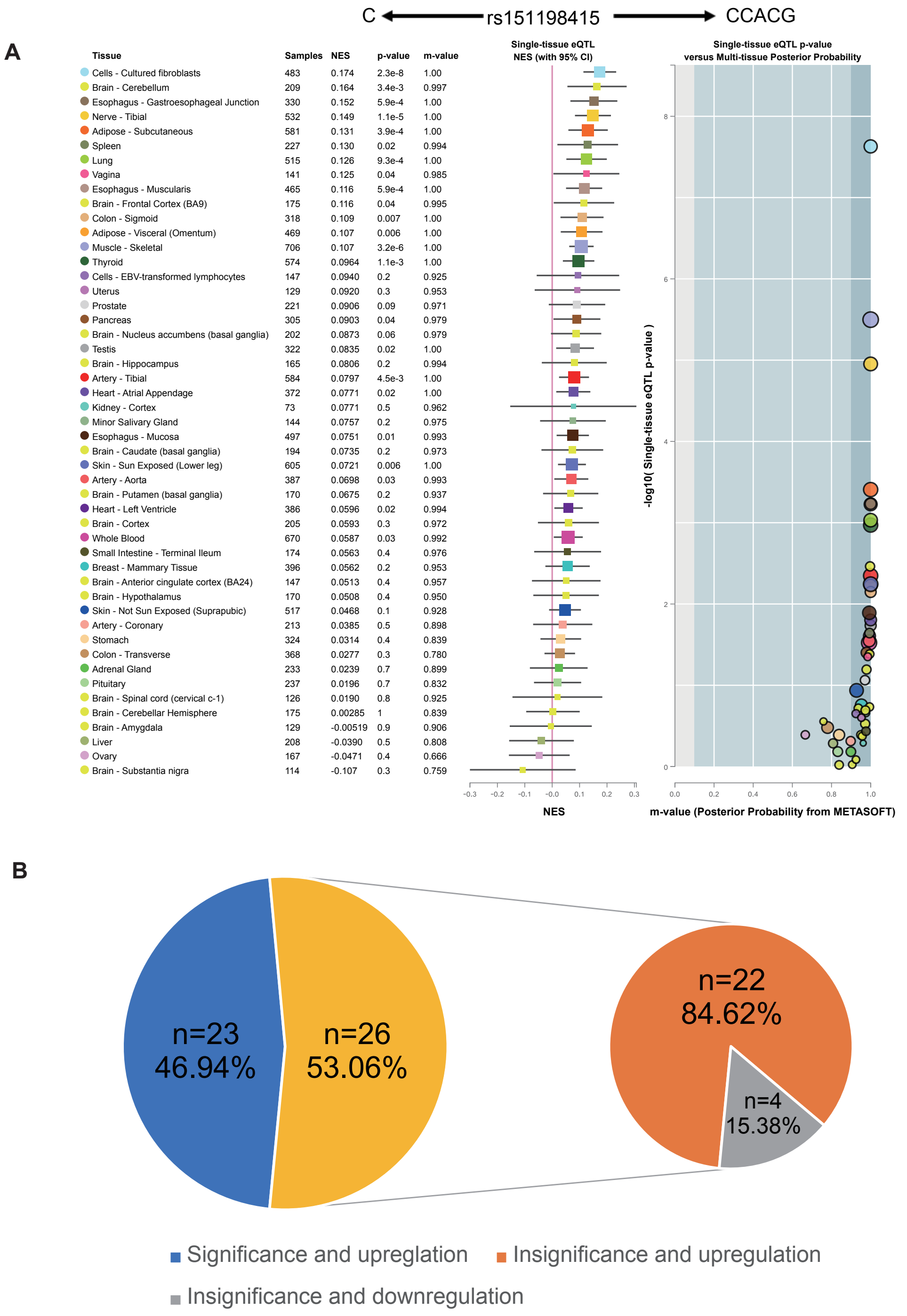

Figure S2: eQTL effect of rs151198415 variant on *ALKBH5* expression in multiple tissues from the GTEx database.

Multi-tissue eQTL comparison plot (**A**); and summary of the significance and directional effect of the rs151198415 variant on *ALKBH5* expression (**B**)

Abbreviations: eQTL, expression quantitative trait loci.

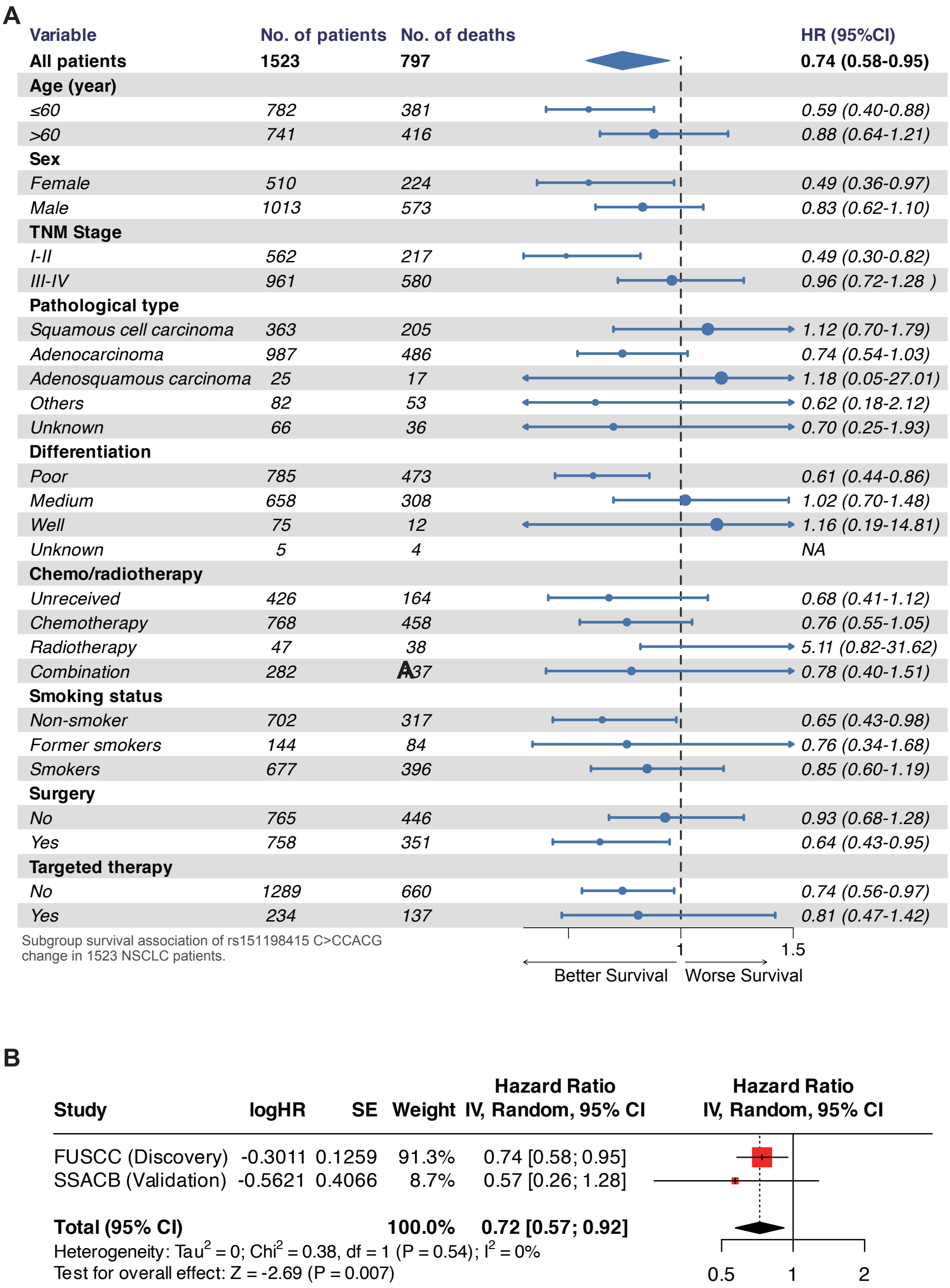

Figure S3: Subgroup analysis and validation of the survival association for the rs151198415 variant in NSCLC patients.

Subgroup survival association for rs151198415 variant in 1,523 NSCLC patients from our ongoing GWAS cohort (**A**); We visulized the association between the rs151198415 variant and survival of NSCLC patients in our FUSCC cohort as discovery and SSACB cohort as validation. Meta-analysis was conducted to pool the

two-stage results (**B**).

Abbreviations: GWAS, genome-wide association study; FUSCC, Our GWAS dataset from Fudan University Shanghai Cancer Center; SSACB, Our GWAS dataset from Shanghai Suburban Adult Cohort and Biobank; CI, Confidence interval.

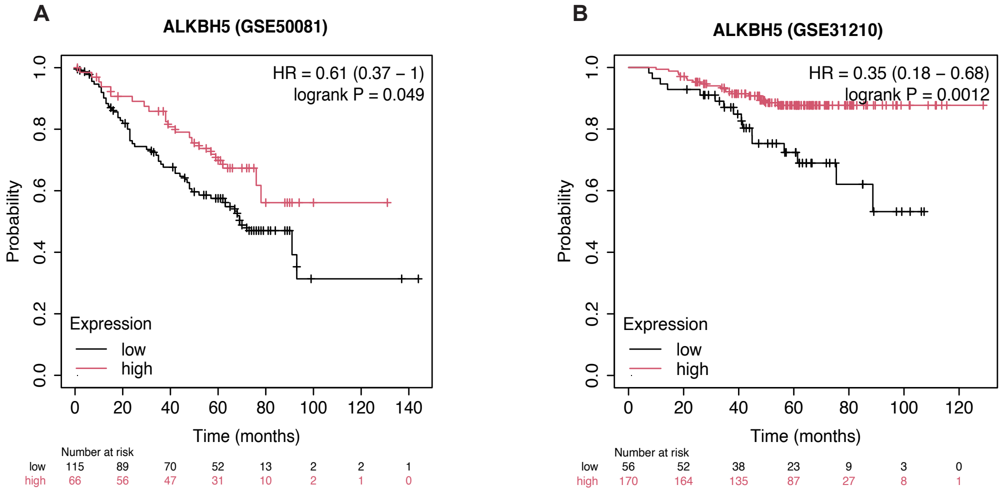

Figure S4: The association between ALKBH5 expression and survival of NSCLC patients in published cohort.

The association of ALKBH5 expression with survival of 181 NSCLC pateints in GSE50081 cohort (**A**) and 226 patients in GSE31210 cohort (**B**)

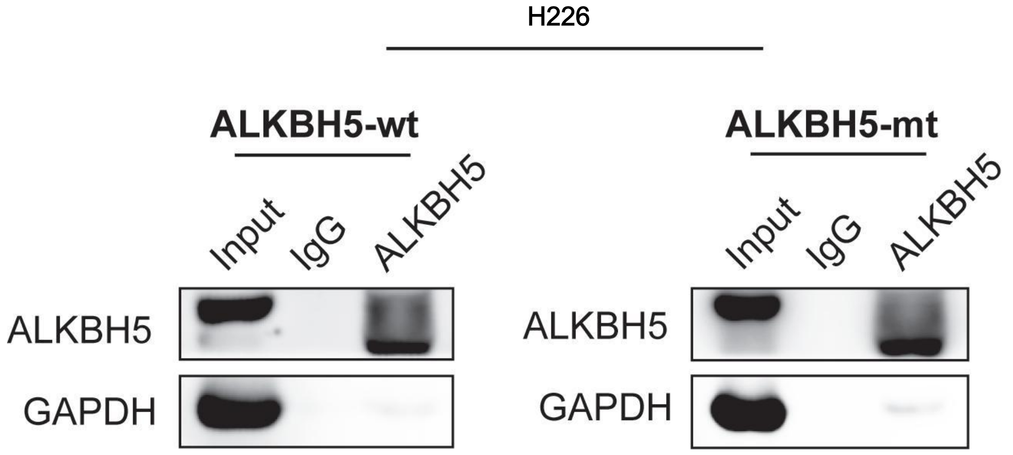

Figure S5: Western blot was performed during the RIP process in H226 cells overexpressing wild-type ALKBH5 and H204A mutant ALKBH5. We detected the expression levels of ALKBH5 and GAPDH for quality control in proteins extracted from input groups, as well as the groups immunoprecipitated by IgG and FBXL5 antibody.

Abbreviations: RIP, RNA immunoprecipitation; wt, Wild; mut, Mutation.

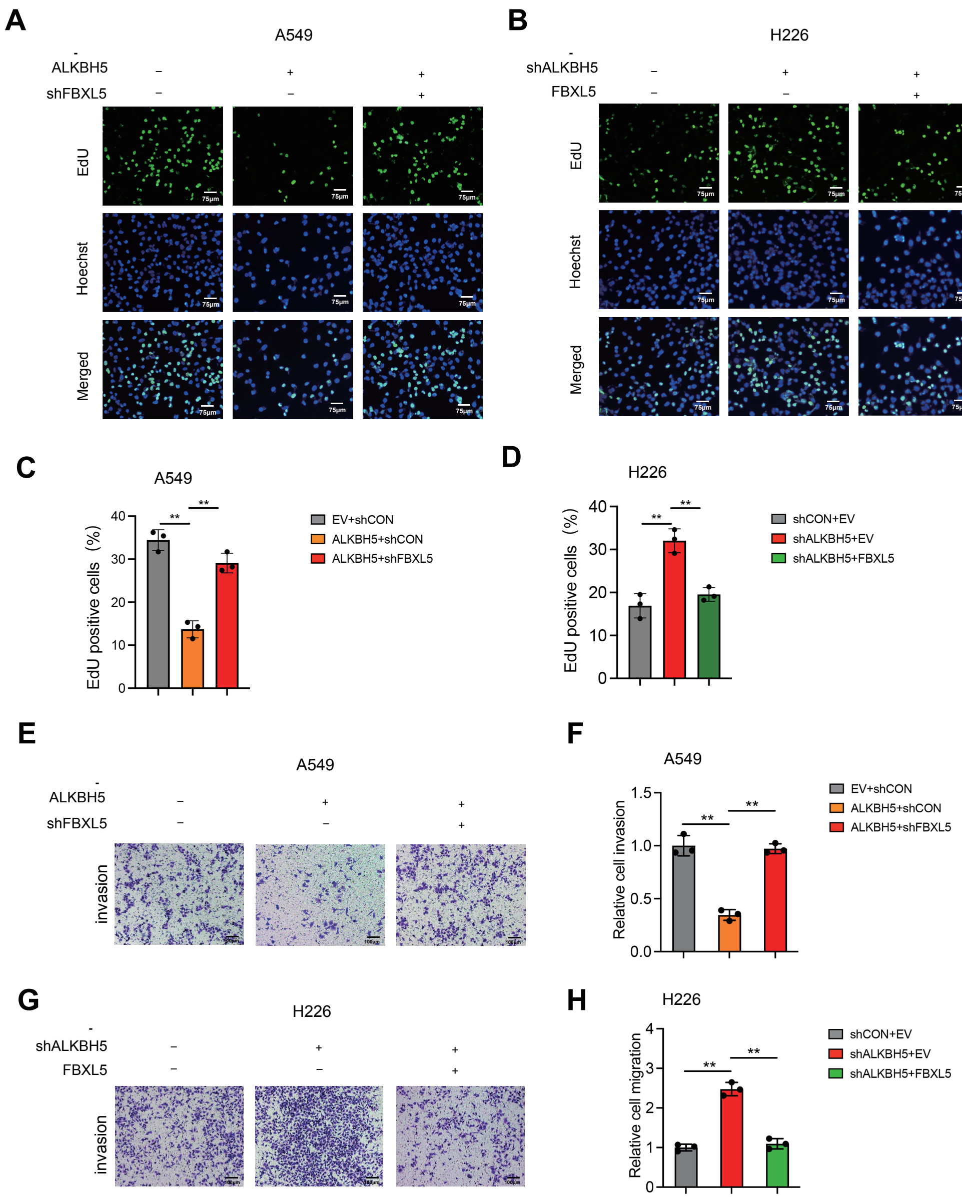

Figure S6: The ALKBH5-FBXL5 axis inhibited proliferation and invasion of NSCLC cells.

The EdU staining in A549 cells was reduced by ALKBH5 overexpression but was restored by FBXL5 knockdown (**A and C**); In H226 cells, the increase in EdU staining due to ALKBH5 knockdown was not sustained in presence of FBXL5 overexpression (**B and D**). ALKBH5 overexpression inhibited the invasion ability of A549 cells, which was restored in the presence of FBXL5 knockdown (**E-F**); ALKBH5 knockdown

promoted the invasion ability, which was interrupted by FBXL5 overexpression in H226 cells (**G-H**).

Abbreviations: EV, empty vector for negative control; shCON, shRNA control group; NSCLC, non-small cell lung carcinoma.

Table S1. m^6A pathway genes included for investigation in present study.

Genes	Type
ALKBH5	Eraser
EIF3A	Reader
EIF4E	Reader
FTO	Eraser
HNRNPA2B1	Reader
HNRNPC	Reader
IGF2BP1	Reader
IGF2BP2	Reader
IGF2BP3	Reader
KIAA1429	Writer
LRPPRC	Writer
METTL3	Writer
METTL4	Writer
METTL14	Writer
METTL16	Writer
RBM15	Writer
RBM15B	Writer
SND1	Reader
WTAP	Writer
YTHDC1	Reader
YTHDC2	Reader
YTHDF1	Reader
YTHDF2	Reader
YTHDF3	Reader
ZC3H13	Writer
DGCR8	Interact with m^6A modification
DICER1	Interact with m^6A modification
DROSHA	Interact with m^6A modification
ADAR	Interact with m^6A modification
ADARB1	Interact with m^6A modification

Table S2. Primer sequence for rs151198415 genotyping using Sanger sequencing and ALKBH5 mRNA expression using qPCR method

	Forward sequence	Reverse sequence
Primer-rs151198415	GGCAGTCCAGGAAA CTCATCA	ATTTTCCACCGCAGCCACTT
Primer-ALKBH5	GACAAGGAAGAGAA CCGGCG	GCATCTTCACCTTTCGGGCA

Table S3. shRNA target sequence for EGR1, ALKBH5, and FBXL5 in present study

**shRNA name**	**Sequence**
Sh-1 (*EGR1*)	GCCAAGCAAACCAATGGTGAT
Sh-2 (*EGR1*)	CGACATCTGTGGAAGAAAGTT
Sh-3 (*EGR1*)	CGGTTACTACCTCTTATCCAT
Sh-1 (*ALKBH5*)	GAAAGGCTGTTGGCATCAATA
Sh-2 (*ALKBH5*)	CCACCCAGCTATGCTTCAGAT
Sh-FBXL5	CCTGATGATGAATGGGTGAAA

Table S4. Sequence for probe prepared for EMSA assay, with unlabeled or biotin-labeled oligonucleotides

Sequence	Type	Sequence
rs151198415 wild	Biotin-labeled probe	GAATGTTACTCCTGCTTAGG GCGTGGCCAGATAGGCTATA TCTGGAGTTTG
rs151198415 mutant	Biotin-labeled probe	GAATGTTACTCCTGCTTAGGG CGTGCGTGGCCAGATAGGCT ATATCTGGAGTTTG
rs151198415 wild	Unlabeled cold probe	GAATGTTACTCCTGCTTAGGG CGTGGCCAGATAGGCTATAT CTGGAGTTTG
rs151198415 mutant	Unlabeled cold probe	GAATGTTACTCCTGCTTAGGG CGTGCGTGGCCAGATAGGCT ATATCTGGAGTTTG

Note: The marked red was the rs151198415 variant. According to the JASPAR database, the underline sequence for rs151198415 mutant prob was the potential motif for EGR1 binding sites .

Table S5. Sequence of taqman fluorescence prob/primers used for taqman CHIP-qPCR and primers in agarosegel electrophoresis

	Forward sequence	Reverse sequence
	**rs151198415**	
Common primer	GCATCATGAATGTTACTCCT	CCAGACCTTCATAAGTCAAG
	**NME1 promoter**	
	GGTGGAGAGAAGAAAGCAAG	TGGGAGTAGGCAGTCATTC
	**CCR5 promoter**	
	TTTCTATTCTCCAAGCACCAG	CTCTTTCAGCAGTCCTTTGTC
	**rs151198415 C**	
	FAM-GCTTAGGGCGTGGCCAGATAGGC-BHQ1	
	**rs151198415 CCACG**	
Fluorescence prob	HEX-CTTAGGGCGTGCGTGGCCAGATAGGC-BHQ1	
	**NME1 promoter**	
	Cy5-GGTCTGAAAAAGCTAGCGCC--BHQ3	
	**CCR5 promoter**	
	ROX-CAGAGACTCCGGTGAACCAA-BHQ2	
	**rs151198415 C**	
	TGTTGTTGTTGTTGTTGTTG	GGACTTGGGCTCTGTGGCTT
	**rs151198415 CCACG**	
Primers used for agarose gel electrophoresis	TGTTGTTGTTGTTGTTGTTGCACG	GGACTTGGGCTCTGTGGCTT
	**NME1 promoter**	
	GGTGGAGAGAAGAAAGCAAG	TGGGAGTAGGCAGTCATTC
	**CCR5 promoter**	
	TTTCTATTCTCCAAGCACCAG	CTCTTTCAGCAGTCCTTTGTC

Abbreviations: qPCR, Quantitative Polymerase Chain Reaction; CHIP, chromatin immunoprecipitation.

Table S6. Sequence for primers used for RIP and MeRIP

**Sequence**	**Gene**	**Forward sequence**	**Reverse sequence**
RIP primer	*FBXL5*	TAGGAAAGATGAAAGTCGTGCT	AGCCATGGAGTAAACGTTTTTC
MeRIP primer	*FBXL5*	AGACATTACCATGCAGTC	CCCTTGTTAGTTAAATCG

Note: The primer for MeRIP was designed based on the *FBXL5* m6A peak sequencing from MeRIP-sequencing

FBXL5 m6A peak sequence, which was used for MeRIP primer design:
TCAACTGCGTGGAAAAATAAAGACATTACCATGCAGTCCACCAAGCAGTATGCC
TGTTTGCACGATTTAACTAACAAGGGCATTGGAGAAGAAATAGATAATGAACACC
CCTGGACTAAGCCTGTTTCTTCTGAGAATTTCACTTCTCCTTATGTGTGGATGTT
AGATGCTGAAGATTTGGCTGATATTGAAGATACTGTGGAATGGAGACATAGAAA
TGTTGAAAGTCTTTGTGTAATGGAAACAGCATCCAACTTTAGTTGTTCCACCTCT
GGTTGTTTTAGTAAGGACATTGTTGGACTAAGGACTAGTGTCTGTTGGCAGCAG
CATTGTGCTTCTCCAGCCTTTGCGTATTGTGGTCACTCATTTTGTTGTACAGGAA
CAGCTTTAAGAACTATGTCATCACTCCCAGAATCTTCTGCAATGTGTAGAAAAGC
AGCAAGGACTAGATTGCCTAGGGGAAAAGACTTAATTTACTTTGGGAGTGAAAA
ATCTGATCAAGAGACTGGACGTGTACTTCTGTTTCT

Table S7. Synonymous mutations designed in FBXL5 CDS

**Potential m^{6}A sites in *FBXL5* CDS region**	**Synonymous mutation sequence in *FBXL5* CDS region**
CGGCATCTTGATCTGTCTGGTTGTGAGA	CGGCATCTTGATCTGTCTGGTTGT
AAATCACAGATGTGGCCCTAGAGAAGAT	GAGAAAATCACAGATGTGGCCCTA
TTCCAGAGCTCTTGGAATTCTGACATCTC	GAGAAGATTTCCAGAGCTCTTGGA
ATCAAAGTGGCTTTTTGAAAACATCTACA	ATTCTGACATCTCATCAAAGTGGC
AGCAAAATTACTTCAACTGCGTGGAAAAA	TTTTTGAAAACATCTACAAGCAAAA
TAAAGACATTACCATGCAGTCCACCAAG	TTACTTCAACTGCGTGGAAAAATA
CAGTATGCCTGTTTGCACGATTTAACTAA	AGGATATCACCATGCAGTCCACCA
CAAGGGCATTGGAGAAGAAATAGATAAT	AGCAGTATGCCTGTTTGCACGATT
GAACACCCCTGGACTAAGCCTGTTTCTT	TGACCAACAAGGGCATTGGAGAA
CTGAGAATTTCACTTCTCCTTATGTGTGG	GAAATAGATAATGAACACCCCTGG
ATGTTAGATGCTGAAGATTTGGCTGATAT	ACCAAGCCTGTTTCTTCTGAGAAT
TGAAGATACTGTGGAATGGAGACATAGA	TTCACTTCTCCTTATGTGTGGATGT
AATGTTGAAAGTCTTTGTGTAATGGAAAC	TAGATGCTGAAGATTTGGCTGATA
AGCATCCAACTTTAGTTGTTCCACCTCTG	TTGAAGATACTGTGGAATGGAGGC
GTTGTTTTAGTAAGGACATTGTTGGACTA	ACAGAAATGTTGAAAGTCTTTGTG
AGGACTAGTGTCTGTTGGCAGCAGCATT	TAATGGAAACAGCATCCAACTTTA
GTGCTTCTCCAGCCTTTGCGTATTGTGG	GTTGTTCCACCTCTGGTTGTTTTA
TCACTCATTTTGTTGTACAGGAACAGCTT	GTAAAGATATCGTTGGACTAAGGA
TAAGAACTATGTCATCACTCCCAGAATCT	CTAGTGTCTGTTGGCAGCAGCATT
TCTGCAATGTGTAGAAAAGCAGCAAGGA	GTGCTTCTCCAGCCTTTGCGTATT
CTAGATTGCCTAGGGGAAAAGACTTAAT	GTGGTCACTCATTTTGTTGTACAG
TTACTTTGGGAGTGAAAAATCTGATCAAG	GTACTGCTTTAAGGACCATGTCAT
AGACTGGACGTGTACTTCTGTTTCTCAGT	CACTCCCAGAATCTTCTGCAATGT
TTATCTGGATGTTATCAGATCACAGACCA	GTAGAAAAGCAGCAAGGACCAGAT
TGGTCTCAGGGTTTTGACTCTGGGAGGA	TGCCTAGGGGAAAGGATTTGATTT
GGGCTGCCTTATTTGGAGCACCTTAATC	ACTTTGGGAGTGAAAAATCTGATC
TCTCTGGTTGTCTTACTATAACTGGTGCA	AAGAAACCGGACGTGTACTTCTGT
GGCCTGCAGGATTTGGTTTCAGCATGTC	TTCTCAGTTTATCTGGATGTTATCA
CTTCTCTGAATGATGAATACTTTTACTAC	GATCACAGACCATGGTCTCAGGGT
TGTGACAACATTAACGGTCCTCATGCTG	TTTGACTCTGGGAGGAGGGCTGC
ATACCGCCAGTGGATGCCAGAATTTGCA	CTTATTTGGAGCACCTTAATCTCTC
GTGTGGTTTTCGAGCCTGCTGCCGCTCT	TGGTTGTCTTACTATAACTGGTGC
GGCGAATGACCCTTGACTTCTGATCTTT	AGGCCTGCAGGATTTGGTTTCAGC
GTCTACTTCATTTAGCTGAGCAGGCTTTC	ATGTCCTTCTCTGAATGATGAATAC
TTTCATGCACTTTACTCATAGCACATTTC	TTTTACTACTGTGACAACATTAACG
TTGTGTTAACCATCCCTTTTTGAGCGTGA	GTCCTCATGCTGATACCGCCAGTG
CTTGTTTTGG	GATGCCAGAATTTGCAGTGTGGTT
	TTCGAGCCTGCTGCCGCTCTGGC
	GAATGACCCTTGACTTCTGATCTTT
	GTCTACTTCATTTAGCTGAGCAGG
	CTTTCTTTCATGCACTTTACTCATA

	GCACATTTCTTGTGTTAACCATCC<br>CTTTTTGAGCGTGACTTGTTTTGG

Note: The potential m^6A sequence was derived from our m^6A sequencing results, based on which we predicted the detailed m^6A site (marked green) using SRAMP website tool. We introduced synonymous mutation (yellow) for the potential m^6A site to construct mutant FBXL5 overexpression vectors.

Abbreviations: m^6A, N6-methyladenosine; CDS, Coding sequence.

Table S9. Clinical demographic of 1523 NSCLC patients from FUSCC cohort included in the present study.

Variable	No. of patients	No. of deaths (%) [a]
Age (year)		
≤60	782	381 (48.7)
>60	741	416 (56.1)
Sex		
Female	510	224 (43.9)
Male	1013	573 (56.6)
TNM Stage		
I-II	562	217 (38.6)
III-IV	961	580 (60.4)
Pathological type		
Squamous cell carcinoma	363	205 (56.5)
Adenocarcinoma	987	486 (49.2)
Adenosquamous carcinoma	25	17 (68.0)
Others	82	53 (64.6)
Unknown	66	36 (54.5)
Differentiation		
Poor	785	473 (60.3)
Medium	658	308 (46.8)
Well	75	12 (16.0)
Unknown	5	4 (80.0)
Chemo/radiotherapy		
Unreceived	426	164 (38.5)
Chemotherapy	768	458 (59.6)
Radiotherapy	47	38 (80.9)
Combination	282	137 (48.6)
Smoking status		
Non-smoker	702	317 (45.2)
Former smokers	144	84 (58.3)
Smokers	677	396 (58.5)
Surgery		
No	765	446 (58.3)
Yes	758	351 (46.3)
Targeted therapy		
No	1289	660 (51.2)
Yes	234	137 (58.5)

Abbreviations: NSCLC, non-small-cell lung carcinoma; TNM, Tumor Node Metastasis.

[a] Death proportion for NSCLC patients with defined clinical demographics

Table S10. The association between number of death-risk alleles and survival of 1523 NSCLC patients.

No. of death-risk alleles	No. of patients	No. of Deaths (%)	Univariate analysis		Multivariate analysis [a]	
			HR (95%CI)	P	HR (95%CI)	P
7-12	214	89 (41.6)	1.00		1.00	
13-15	742	374 (50.4)	**1.39 (1.10-1.75)**	**0.005**	**1.53 (1.21-1.93)**	**4×10^{-4}**
16-21	567	334 (58.9)	**1.77 (1.40-2.24)**	**1.62×10^{-6}**	**2.19 (1.72-2.77)**	**1.07×10^{-10}**
P_{trend}						9.71×10^{-12}

[a] Adjusted for age, sex, tumor differentiation, pathology type, TNM stage, smoking status and treatment of the NSCLC patients.

Abbreviations: NSCLC, Non-small cell lung carcinoma; *P*, *P* value; HR, hazards ratio; We marked the results with statistically significance in **bold**.

Table S11. Functional prediction for survival-associated SNPs with RegulomeDB database

chrom	start	end	rsids	probability	ranking	ChIP	Chromatin_accessibility	Footprint	Footprint_matched	IC_matched_max	IC_max	PWM	PWM_matched	QTL
chr17	17838182	17838183	rs151198415	0.83	1a	TRUE	TRUE	TRUE	TRUE	0	1.82	TRUE	TRUE	TRUE
chr16	53794153	53794154	rs17817964	0.18917	1b	TRUE	TRUE	TRUE	FALSE	0	0.95	TRUE	FALSE	TRUE
chr16	53842949	53842950	rs7204450	0.19833	1b	TRUE	TRUE	TRUE	FALSE	0	0.21	TRUE	FALSE	TRUE
chr17	17838560	17838561	rs7359509	0.94346	1b	TRUE	TRUE	TRUE	TRUE	0	1.56	TRUE	FALSE	TRUE
chr17	17973781	17973782	rs11870660	0.44867	1b	TRUE	TRUE	TRUE	FALSE	0	0.67	TRUE	FALSE	TRUE
chr17	18009742	18009743	rs6502632	0.64002	1b	TRUE	TRUE	TRUE	FALSE	0	0.34	TRUE	FALSE	TRUE
chr17	18064082	18064083	rs2955371	0.90197	1b	TRUE	TRUE	TRUE	FALSE	0	0.4	TRUE	FALSE	TRUE
chr17	17985234	17985235	rs4459604	0.645	1d	TRUE	TRUE	FALSE	FALSE	0	0.93	TRUE	FALSE	TRUE
chr17	18046848	18046849	rs12939020	0.33833	1d	TRUE	TRUE	FALSE	FALSE	0	1.88	TRUE	FALSE	TRUE
chr14	21510150	21510151	rs719785	0.55324	1f	TRUE	FALSE	FALSE	FALSE	0	0	FALSE	FALSE	TRUE
chr16	53775210	53775211	rs55872725	0.55436	1f	TRUE	TRUE	FALSE	FALSE	0	0	FALSE	FALSE	TRUE
chr16	53842629	53842630	rs7203883	0.55324	1f	TRUE	FALSE	FALSE	FALSE	0	0	FALSE	FALSE	TRUE
chr16	53842653	53842654	rs7205213	0.55324	1f	TRUE	FALSE	FALSE	FALSE	0	0	FALSE	FALSE	TRUE
chr16	53842694	53842695	rs7204060	0.55324	1f	TRUE	FALSE	FALSE	FALSE	0	0	FALSE	FALSE	TRUE
chr16	53843129	53843130	rs2388406	0.55436	1f	TRUE	TRUE	FALSE	FALSE	0	0	FALSE	FALSE	TRUE
chr16	54063264	54063265	rs2689246	0.55436	1f	TRUE	TRUE	FALSE	FALSE	0	0	FALSE	FALSE	TRUE
chr16	54066456	54066457	rs1108086	0.55436	1f	TRUE	TRUE	FALSE	FALSE	0	0	FALSE	FALSE	TRUE
chr17	17506394	17506395	rs1918249	0.66703	1f	TRUE	TRUE	TRUE	FALSE	0	0	FALSE	FALSE	TRUE
chr17	17821474	17821475	rs11656665	0.55324	1f	TRUE	FALSE	FALSE	FALSE	0	0	FALSE	FALSE	TRUE
chr17	17832637	17832638	rs4924821	0.55436	1f	TRUE	TRUE	FALSE	FALSE	0	0	FALSE	FALSE	TRUE
chr17	17836849	17836850	rs60282872	0.55436	1f	TRUE	TRUE	FALSE	FALSE	0	0	FALSE	FALSE	TRUE
chr17	17836966	17836967	rs13306736	0.55436	1f	TRUE	TRUE	FALSE	FALSE	0	0	FALSE	FALSE	TRUE
chr17	17838354	17838355	rs12946746	0.55436	1f	TRUE	TRUE	FALSE	FALSE	0	0	FALSE	FALSE	TRUE
chr17	17971171	17971172	rs8068175	0.55324	1f	TRUE	FALSE	FALSE	FALSE	0	0	FALSE	FALSE	TRUE
chr17	17972027	17972028	rs57728924	0.55436	1f	TRUE	TRUE	FALSE	FALSE	0	0	FALSE	FALSE	TRUE
chr17	17972092	17972093	rs59304093	0.55436	1f	TRUE	TRUE	FALSE	FALSE	0	0	FALSE	FALSE	TRUE
chr17	17973130	17973131	rs28366006	0.55436	1f	TRUE	TRUE	FALSE	FALSE	0	0	FALSE	FALSE	TRUE
chr17	17974844	17974845	rs4299203	0.55324	1f	TRUE	FALSE	FALSE	FALSE	0	0	FALSE	FALSE	TRUE
chr17	17984362	17984363	rs6146007	0.22271	1f	FALSE	TRUE	FALSE	FALSE	0	0	FALSE	FALSE	TRUE
chr17	17993244	17993245	rs9911850	0.18086	1f	FALSE	TRUE	TRUE	FALSE	0	1.84	TRUE	FALSE	TRUE
chr17	17993358	17993359	rs9912096	0.22271	1f	FALSE	TRUE	FALSE	FALSE	0	0	FALSE	FALSE	TRUE
chr17	17994424	17994425	rs4924832	0.55436	1f	TRUE	TRUE	FALSE	FALSE	0	0	FALSE	FALSE	TRUE
chr17	17996306	17996307	rs6502631	0.55436	1f	TRUE	TRUE	FALSE	FALSE	0	0	FALSE	FALSE	TRUE
chr17	17998631	17998632	rs8075189	0.55436	1f	TRUE	TRUE	FALSE	FALSE	0	0	FALSE	FALSE	TRUE
chr17	18000190	18000191	rs10558401	0.55436	1f	TRUE	TRUE	FALSE	FALSE	0	0	FALSE	FALSE	TRUE
chr17	18001974	18001975	rs58365097	0.55436	1f	TRUE	TRUE	FALSE	FALSE	0	0	FALSE	FALSE	TRUE

chr17	18003205	18003206	rs62072049	0.22271	1f	FALSE	TRUE	FALSE	FALSE	0	0	FALSE	FALSE	TRUE
chr17	18007699	18007700	rs12150369	0.55436	1f	TRUE	TRUE	FALSE	FALSE	0	0	FALSE	FALSE	TRUE
chr17	18008696	18008697	rs9897761	0.55436	1f	TRUE	TRUE	FALSE	FALSE	0	0	FALSE	FALSE	TRUE
chr17	18009233	18009234	rs4365348	0.55436	1f	TRUE	TRUE	FALSE	FALSE	0	0	FALSE	FALSE	TRUE
chr17	18010189	18010190	rs7212167	0.55436	1f	TRUE	TRUE	FALSE	FALSE	0	0	FALSE	FALSE	TRUE
chr17	18012476	18012477	rs7223696	0.22271	1f	FALSE	TRUE	FALSE	FALSE	0	0	FALSE	FALSE	TRUE
chr17	18018965	18018966	rs8069811	0.55436	1f	TRUE	TRUE	FALSE	FALSE	0	0	FALSE	FALSE	TRUE
chr17	18020745	18020746	rs8079418	0.22271	1f	FALSE	TRUE	FALSE	FALSE	0	0	FALSE	FALSE	TRUE
chr17	18026938	18026939	rs2955378	0.22271	1f	FALSE	TRUE	FALSE	FALSE	0	0	FALSE	FALSE	TRUE
chr17	18038049	18038050	rs2955383	0.66703	1f	TRUE	TRUE	TRUE	FALSE	0	0	FALSE	FALSE	TRUE
chr17	18043086	18043087	rs2955357	0.55436	1f	TRUE	TRUE	FALSE	FALSE	0	0	FALSE	FALSE	TRUE
chr17	18043308	18043309	rs2955358	0.55436	1f	TRUE	TRUE	FALSE	FALSE	0	0	FALSE	FALSE	TRUE
chr17	18044395	18044396	rs2955382	0.55436	1f	TRUE	TRUE	FALSE	FALSE	0	0	FALSE	FALSE	TRUE
chr17	18045160	18045161	rs2955355	0.22271	1f	FALSE	TRUE	FALSE	FALSE	0	0	FALSE	FALSE	TRUE
chr17	18046487	18046488	rs12948749	0.55324	1f	TRUE	FALSE	FALSE	FALSE	0	0	FALSE	FALSE	TRUE
chr17	18049124	18049125	rs11652894	0.70294	1f	FALSE	TRUE	TRUE	FALSE	0	2	TRUE	FALSE	TRUE
chr17	18049687	18049688	rs4925136	0.55436	1f	TRUE	TRUE	FALSE	FALSE	0	0	FALSE	FALSE	TRUE
chr17	18055087	18055088	rs8082590	0.22271	1f	FALSE	TRUE	FALSE	FALSE	0	0	FALSE	FALSE	TRUE
chr17	18058034	18058035	rs2955368	0.22271	1f	FALSE	TRUE	FALSE	FALSE	0	0	FALSE	FALSE	TRUE
chr17	18061030	18061031	rs9894138	0.22271	1f	FALSE	TRUE	FALSE	FALSE	0	0	FALSE	FALSE	TRUE
chr17	18061402	18061403	rs2955356	0.55436	1f	TRUE	TRUE	FALSE	FALSE	0	0	FALSE	FALSE	TRUE
chr17	18063209	18063210	rs34519174	0.55436	1f	TRUE	TRUE	FALSE	FALSE	0	0	FALSE	FALSE	TRUE
chr21	45266578	45266579	rs6518220	0.55436	1f	TRUE	TRUE	FALSE	FALSE	0	0	FALSE	FALSE	TRUE
chr21	45266583	45266584	rs6518221	0.55436	1f	TRUE	TRUE	FALSE	FALSE	0	0	FALSE	FALSE	TRUE
chr21	45297788	45297789	rs4819056	0.55324	1f	TRUE	FALSE	FALSE	FALSE	0	0	FALSE	FALSE	TRUE
chr8	94482833	94482834	rs1992371	0.55324	1f	TRUE	FALSE	FALSE	FALSE	0	0	FALSE	FALSE	TRUE
chr8	94530643	94530644	rs56716157	0.55324	1f	TRUE	FALSE	FALSE	FALSE	0	0	FALSE	FALSE	TRUE
chr8	94556760	94556761	rs60370483	0.55324	1f	TRUE	FALSE	FALSE	FALSE	0	0	FALSE	FALSE	TRUE
chr17	18004078	18004079	rs7209003	0.51392	7	FALSE	FALSE	FALSE	FALSE	0	0	FALSE	FALSE	TRUE
chr17	18016434	18016435	rs9913277	0.51392	7	FALSE	FALSE	FALSE	FALSE	0	0	FALSE	FALSE	TRUE
chr17	18046642	18046643	rs7210400	0.51392	7	FALSE	FALSE	FALSE	FALSE	0	0	FALSE	FALSE	TRUE
chr17	18057298	18057299	rs11650021	0.51392	7	FALSE	FALSE	FALSE	FALSE	0	0	FALSE	FALSE	TRUE
chr5	113483329	113483330	rs348963	0.51392	7	FALSE	FALSE	FALSE	FALSE	0	0	FALSE	FALSE	TRUE

Note: The marked red were predicted functional SNPs with Regulome DB ranking score 1a to 1b

Table S12.The survival association of rs151198415 variant in 1523 NSCLC patients

**Locus/ rs#**	**Alleles**	**Univariate analysis**		**Multivariate analysis**			
		**HR (95% CI)**	***P***	**HR (95% CI)** [a]	**$FPRP_{0.1}$**	**$BFDP_{0.1}$**	***P***
chr17:17838183 (GRCh38)/rs151198415	C>CCACG	**0.71 (0.56-0.91)**	**0.006**	**0.74 (0.58-0.95)**	0.171	0.692	0.019

[a] Adjusted for age, sex, tumor differentiation, pathology type, TNM stage, smoking status and treatment of the NSCLC patients.

Abbreviations: NSCLC, Non-small lung cell carcinoma; rs#, reference ID for target SNP; HR, hazards ratio; SNP, single nucleotide polymorphisms; BFDP, Bayesian false-discovery probability; FPRP, False positive report probability; *P*, *P* value. Significant survival association were marked in **bold**.

Table S13. Clinical demographic of 237 NSCLC patients included inthe validation from the SSACB prospective cohort

Variable	No. of patients	No. of deaths (%) [a]
Age (year)		
≤60	40	2 (5.0)
>60	197	64 (32.5)
Sex		
Female	115	14 (12.2)
Male	122	52 (42.6)
Surgery		
No	61	50 (82.0)
Yes	176	16 (9.1)
Chemo/radiotherapy		
Unreceived	166	37 (22.3)
Received	71	29 (40.8)
Smoking		
No	169	34 (20.1)
Yes	68	32 (47.1)
Drinking		
No	184	40 (21.7)
Yes	53	26 (49.1)
COPD history		
No	232	65 (28.0)
Yes	5	1 (20.0)

Abbreviations: NSCLC, non-small-cell lung carcinoma.

[a] Death proportion for NSCLC patients with defined clinical demographics

Table S14. Significant m^6A modification and gene regulation by ALKBH5 overexpression in A549 cells

geneName	Sequence NO.	lg.fdr for m6A regulation	log2(fc) for m6A regulation	m6A_regulation	qval for gene regulation	log2(fc) for gene regulation	gene_regulation
EPHB2	41	-2.38	-0.398	down	1.03325E-07	0.799155199	up
PTPRF	82	-7.44	0.424	up	0.025075315	0.420300322	up
PTPRF	83	-8.5	-0.361	down	0.025075315	0.420300322	up
PTPRF	84	-3.67	-0.56	down	0.025075315	0.420300322	up
KIRREL1	158	-1.55	0.636	up	5.59349E-06	0.646778099	up
KIRREL1	159	-6.24	-0.377	down	5.59349E-06	0.646778099	up
KIRREL1	160	-1.45	0.867	up	5.59349E-06	0.646778099	up
NDUFS2	161	-6.19	0.356	up	0.016539793	-0.376055284	down
DTL	195	-2.83	2.83	up	0.003839428	0.651297786	up
MAP3K21	214	-5.29	-0.392	down	0.037352201	0.436300375	up
RAP1GAP	266	-6.49	0.443	up	5.40318E-16	-1.179263992	down
DHCR24	331	-6.05	0.503	up	0.011627636	-0.428248877	down
ATOH8	541	-3.79	-0.601	down	0.000217872	-1.057195777	down
NRP2	604	-1.51	-0.28	down	0.003345746	0.428474795	up
NRP2	605	-3.11	-0.607	down	0.003345746	0.428474795	up
NRP2	606	-2.99	-0.505	down	0.003345746	0.428474795	up
NRP2	607	-3.38	-0.327	down	0.003345746	0.428474795	up
C2orf72	622	-8.14	-1.17	down	0.000699068	-0.768147991	down
KIF3C	657	-6.68	-0.277	down	0.000130001	0.618819653	up
LYPD1	731	-31.9	-0.554	down	2.85252E-05	0.911788592	up
NOSTRIN	735	-2.94	-0.791	down	0.024391663	-1.053192218	down
NRP2	754	-1.41	-0.39	down	0.003345746	0.428474795	up
SH3BP5	931	-2.3	0.871	up	0.02024322	-0.446333171	down
PRICKLE2	987	-1.41	-0.372	down	0.021279946	0.421427096	up
DCBLD2	993	-9.23	-0.244	down	0.010083727	0.405147935	up
DCBLD2	994	-7.88	0.447	up	0.010083727	0.405147935	up
MYLK	1005	-4.17	1.46	up	0.030447413	0.390119697	up
MYLK	1006	-2.16	-0.672	down	0.030447413	0.390119697	up
MYLK	1007	-2.23	-0.368	down	0.030447413	0.390119697	up
PLOD2	1021	-8.24	-0.3	down	0.00262959	0.461315197	up
DOK7	1073	-2.45	-0.469	down	0.013427207	-1.328829947	down
FBXL5	1154	-30.9	-1.59	down	7.40631E-08	0.812496944	up
FBXL5	1155	-89.9	-1.57	down	7.40631E-08	0.812496944	up
PPARGC1A	1159	-1.7	-0.288	down	0.00201967	-0.512795542	down
HMGB2	1188	-3.34	0.336	up	0.03591291	0.456795402	up

TRIO	1205	-23.7	-1.01	down	0.002867975	0.52948264	up
TRIO	1206	-15.6	-0.434	down	0.002867975	0.52948264	up
LMNB1	1235	-4.98	0.556	up	0.012458376	0.521740057	up
SLC12A2	1237	-3.48	-0.24	down	0.006373589	-0.426583607	down
LPCAT1	1321	-12.6	-0.251	down	0.041700101	0.343399607	up
LPCAT1	1322	-2.08	-0.236	down	0.041700101	0.343399607	up
ANKH	1327	-4.11	0.288	up	0.029853575	-0.416448801	down
AL365205	1476	-2.36	2.06	up	9.12523E-05	-2.680636557	down
OGFRL1	1494	-2.03	-0.356	down	0.03579545	0.535941701	up
DCBLD1	1510	-2.86	-0.581	down	7.20708E-06	0.735111572	up
DCBLD1	1511	-10.8	-0.464	down	7.20708E-06	0.735111572	up
ZSCAN16-AS1	1564	-4.49	0.777	up	0.002083411	-0.984178465	down
MCM3	1611	-12.8	-0.433	down	0.041700101	0.403331142	up
RSPO3	1638	-1.7	-0.366	down	0.00010732	0.597152025	up
EGFR	1701	-8.48	-0.275	down	0.003851445	0.447184732	up
SERPINE1	1754	-4.57	0.427	up	3.29509E-23	1.42859498	up
CYTH3	1811	-4.44	-0.222	down	0.047840411	0.353471995	up
TNS3	1835	-3.13	-0.241	down	0.006373589	0.414448641	up
TNS3	1836	-44.6	-0.381	down	0.006373589	0.414448641	up
GPR37	1882	-3.49	0.18	up	0.000216976	-0.668125875	down
PLEKHA2	1953	-2.95	1.08	up	0.001756558	0.600513897	up
TRIB1	1988	-2.09	-0.132	down	0.043305718	0.383161979	up
LY6E	1993	-6.63	0.424	up	0.045687594	-0.42952729	down
ATP8B5P	2137	-4.44	-0.301	down	0.000118324	-2.725546486	down
DAPK1	2154	-35.3	-0.364	down	0.014336564	0.383225217	up
CTSL	2155	-10.5	0.272	up	0.008181014	-0.49135217	down
AOPEP	2160	-6.79	-1.1	down	0.049618264	0.359066998	up
OLFML2A	2178	-4.7	-0.431	down	0.004084292	0.571970144	up
NCS1	2202	-5.69	-0.351	down	0.009115631	0.495643675	up
BAG1	2243	-1.67	0.272	up	0.025914716	-0.398742587	down
AQP3	2244	-11.5	0.349	up	2.2349E-08	-0.774343493	down
PFKFB3	2360	-7.89	-0.233	down	0.024210121	0.355027755	up
PFKFB3	2361	-15.4	-0.183	down	0.024210121	0.355027755	up
BICC1	2393	-12.6	-0.392	down	0.0192049	0.431285626	up
VCL	2402	-4.1	-0.292	down	0.000521218	0.527065759	up
SEMA4G	2433	-4.39	0.643	up	7.43712E-07	-0.774552071	down
SEMA4G	2434	-1.33	-0.306	down	7.43712E-07	-0.774552071	down

CNNM2	2441	-2.32	-0.253	down	0.030102242	-0.489131271	down
SLC25A28	2535	-55.4	-1.08	down	0.00050507	0.733204181	up
AFAP1L2	2552	-3.45	-0.471	down	0.000148978	0.75187216	up
MKI67	2567	-12.7	-0.222	down	0.001340823	0.633871021	up
MKI67	2568	-3.98	-0.452	down	0.001340823	0.633871021	up
MKI67	2569	-1.38	-0.26	down	0.001340823	0.633871021	up
ADM	2590	-4.3	-0.554	down	0.009486617	0.539669988	up
MICAL2	2595	-2.34	-0.877	down	0.02888671	0.416785641	up
NDUFV1	2685	-13.4	0.373	up	0.04221408	-0.352457899	down
ST3GAL4	2745	-6.13	0.515	up	0.000248252	-0.691876467	down
H19	2757	-3.34	0.743	up	1.55854E-05	-0.846520275	down
ARRB1	2835	-2.36	-0.46	down	0.032313576	-0.435789231	down
FXYD2	2854	-16.6	0.284	up	0.001876637	-0.541845231	down
FXYD2	2855	-23.4	0.276	up	0.001876637	-0.541845231	down
KIRREL3	2866	-1.9	0.582	up	0.01360055	1.02789957	up
RIMKLB	2900	-6.23	1.12	up	0.032878458	-0.508997266	down
COX6A1	2981	-52.3	0.293	up	0.029002816	-0.366412448	down
AC023055	3080	-5.81	-0.219	down	3.33031E-06	5.753933714	up
ANKLE2	3153	-12.4	-0.693	down	5.25938E-07	0.693538583	up
ANKLE2	3154	-2.55	-0.655	down	5.25938E-07	0.693538583	up
ANKLE2	3155	-21	-0.874	down	5.25938E-07	0.693538583	up
ANKLE2	3156	-5.5	-0.98	down	5.25938E-07	0.693538583	up
ANKLE2	3157	-2.2	0.599	up	5.25938E-07	0.693538583	up
TSC22D1	3210	-6.37	0.295	up	0.037429613	-0.365344399	down
ZNF219	3227	-2.42	1.18	up	0.024641926	-0.444911996	down
ZNF219	3315	-2.25	1.15	up	0.024641926	-0.444911996	down
AJUBA	3319	-4.47	-0.324	down	0.000589871	0.535617993	up
BMP4	3343	-8.91	1.03	up	0.034765314	0.481572093	up
JAG2	3407	-3.7	-0.342	down	0.005264124	0.57895321	up
EHD4	3506	-1.34	0.629	up	0.004565231	0.560958489	up
NTRK3	3569	-10.5	-0.806	down	3.63613E-17	1.183438577	up
CACNA1H	3594	-10.4	-1.24	down	0.00088363	-0.618794325	down
ADCY7	3715	-1.79	-0.94	down	0.000711554	0.634880969	up
ADCY7	3716	-20.7	-0.692	down	0.000711554	0.634880969	up
USP10	3762	-64.7	-0.613	down	0.006073141	0.419208395	up
MTSS2	3881	-22.5	-0.448	down	0.049248984	0.38738893	up
RFWD3	3886	-3.17	-0.382	down	0.024645777	0.436332345	up

TUBB3	3909	-1.58	-0.34	down	0.031940241	0.37130625	up
PIMREG	3934	-1.76	-0.705	down	0.039832492	0.568317721	up
ALKBH5	3965	-9.85	-0.267	down	4.78193E-25	1.240761934	up
ALKBH5	3966	-87.9	1.21	up	4.78193E-25	1.240761934	up
SLC9A3R1	4079	-1.95	0.32	up	0.000347889	-0.54431458	down
SLC9A3R1	4080	-1.73	0.257	up	0.000347889	-0.54431458	down
NXN	4119	-11.3	-0.363	down	0.004101857	0.616714759	up
JUP	4216	-10.7	0.46	up	0.000607737	-0.551748439	down
CAVIN1	4221	-3.32	0.405	up	0.000380326	0.565976244	up
MTCL1	4321	-6.85	-0.827	down	0.013006709	0.435612139	up
MTCL1	4322	-7.23	-0.346	down	0.013006709	0.435612139	up
MTCL1	4323	-2.25	-0.404	down	0.013006709	0.435612139	up
MTCL1	4324	-23.6	-0.429	down	0.013006709	0.435612139	up
MTCL1	4325	-3.58	-0.625	down	0.013006709	0.435612139	up
MTCL1	4326	-1.71	0.78	up	0.013006709	0.435612139	up
TUBB6	4331	-2.06	-0.326	down	0.003934611	0.557230274	up
ALPK2	4384	-1.76	-0.469	down	0.008181014	0.517735711	up
ALPK2	4386	-7.64	-0.542	down	0.008181014	0.517735711	up
TJP3	4432	-2.46	0.566	up	0.006104745	-0.65516032	down
CHAF1A	4434	-22.5	-0.459	down	0.005620934	0.594349546	up
CHAF1A	4436	-4.43	-0.602	down	0.005620934	0.594349546	up
CYP4F3	4464	-3.64	-0.407	down	0.007656948	-0.767482924	down
IFI30	4483	-3.57	0.397	up	0.004350335	-0.800507658	down
ZNF431	4498	-1.49	-1.31	down	0.025792214	0.988371059	up
AD000671	4517	-10.8	0.448	up	9.28554E-05	inf	up
CEACAM6	4551	-3.87	-0.12	down	3.72535E-14	-0.836079501	down
CD320	4683	-3.2	0.32	up	0.035267837	-0.451000556	down
FCGBP	4750	-3.63	-0.769	down	6.39005E-14	-1.349255263	down
FCGBP	4751	-15.1	-0.757	down	6.39005E-14	-1.349255263	down
FCGBP	4752	-3.27	-0.632	down	6.39005E-14	-1.349255263	down
ZNF611	4807	-1.52	-2.1	down	0.009486617	1.538684267	up
CST1	4939	-2.49	0.653	up	5.78029E-05	-0.912164262	down
JPH2	4965	-1.33	-0.23	down	0.017574651	1.243433981	up
FLNA	5316	-43	0.326	up	0.004541559	0.433246328	up
MYORG	5360	-2.05	0.641	up	0.008613552	-0.504996513	down
GINS3	5398	-7.98	-0.612	down	0.045548722	0.530868344	up
